# Benefits and Challenges in the Incorporation of Insects in Food Products

**DOI:** 10.3389/fnut.2021.687712

**Published:** 2021-06-30

**Authors:** Beatriz A. Acosta-Estrada, Alicia Reyes, Cristina M. Rosell, Dolores Rodrigo, Celeste C. Ibarra-Herrera

**Affiliations:** ^1^Tecnologico de Monterrey, Escuela de Ingeniería y Ciencias, Centro de Biotecnología-FEMSA, Monterrey, Mexico; ^2^Tecnologico de Monterrey, Escuela de Ingeniería y Ciencias, Departamento de Bioingeniería, Puebla, Mexico; ^3^Instituto de Agroquimica y Tecnologia de Alimentos (IATA-CSIC), Valencia, Spain

**Keywords:** edible insect, bioactive compounds, consumer attitude, food safety, food processing

## Abstract

Edible insects are being accepted by a growing number of consumers in recent years not only as a snack but also as a side dish or an ingredient to produce other foods. Most of the edible insects belong to one of these groups of insects such as caterpillars, butterflies, moths, wasps, beetles, crickets, grasshoppers, bees, and ants. Insect properties are analyzed and reported in the articles reviewed here, and one common feature is nutrimental content, which is one of the most important characteristics mentioned, especially proteins, lipids, fiber, and minerals. On the other hand, insects can be used as a substitute for flour of cereals for the enrichment of snacks because of their high content of proteins, lipids, and fiber. Technological properties are not altered when these insects-derived ingredients are added and sensorial analysis is satisfactory, and only in some cases, change in color takes place. Insects can be used as substitute ingredients in meat products; the products obtained have higher mineral content than traditional ones, and some texture properties (like elasticity) can be improved. In extruded products, insects are an alternative source of proteins to feed livestock, showing desirable characteristics. Isolates of proteins of insects have demonstrated bioactive activity, and these can be used to improve food formulations. Bioactive compounds, as antioxidant agents, insulin regulators, and anti-inflammatory peptides, are high-value products that can be obtained from insects. Fatty acids that play a significant role in human health and lipids from insects have showed positive impacts on coronary disease, inflammation, and cancer. Insects can be a vector for foodborne microbial contamination, but the application of good manufacturing practices and effective preservation techniques jointly with the development of appropriate safety regulations will decrease the appearance of such risks. However, allergens presented in some insects are a hazard that must be analyzed and taken into account. Despite all the favorable health-promoting characteristics present in insects and insects-derived ingredients, willingness to consume them has yet to be generalized.

## Introduction

In the latest years, researchers are looking at insects as a potential source of alternative proteins. It is clear that the exponential growth of the populations creates the societal challenge of ensuring their appropriate feeding, resulting in the increment of the global demand for meat. Meat production has been discussed as a non-sustainable process. The livestock sector uses 2/3 of all the agricultural land (1) and is responsible for more than 14% of total greenhouse gas emissions and 59% of total agricultural ammonia emissions (2). Nowadays, around the world, more than 2,000 insect species are consumed mainly in tropical countries, namely, beetles (coleoptera); caterpillars, butterflies, moths (lepidoptera); wasps, bees, and ants (hymenoptera); crickets, grasshoppers, and locusts (orthoptera); cicadas, honey ants, aphids, plant hoppers, leafhoppers, scale insects, and true bugs (Hemiptera); and others such as termites and cockroaches (3). Therefore, edible insects have been the focus of several researchers, documenting that edible insects contain essential nutrients such as proteins, vitamins, minerals, and fatty acids. The protein content is especially high, reaching around 77% in many species (4). Insects are a source of high-value proteins; their essential amino acid score (the percentage of essential amino acids required in an ideal protein) ranges between 46 and 96% (5). In addition, insects are also high in minerals like copper (Cu), selenium (Se), iron (Fe), zinc (Zn), calcium (Ca), magnesium (Mg), manganese (Mn), and phosphorus (P), as well as vitamins like biotin, riboflavin, pantothenic acid, and folic acid (6). Then, insects represent a great option as an alternative protein source mainly due to their high content of proteins reported, and their low environmental impact in terms of low emission of greenhouse gases (7), the yield of production in terms of the amount of land required to produce one kilogram of protein from insects (7), as well as their high-feed-conversion efficiencies (3). Edible species can be eaten as larvae or in adult form, and both have showed a high feed conversion ratio (FCR) as their production requires a reduced amount of water, energy, and land. All these factors mentioned before can help to reduce the ammonia emissions to environment (8). In this scenario, insects are seen as one of the main future sources of proteins for human consumption or as pets and fish food and livestock feed.

Insects can be used as a food ingredient to increase the nutritional content of different meals around the world, with specific purposes such as the improvement of the nutrition intake of the population in countries where access to a protein source is scarce; to offer products with high-protein content that complement human diet, addressing the increasing demand for alternative sources of protein or to develop exotic food products to attract the attention of culinary tourism. Different products containing insects or parts of them are being commercialized worldwide, such as in cereal-based foods, animal-derived protein foods, animal feed, and other products like sauces and honey spread (9–12). In addition to nutritional benefits, the findings of some studies show that insects have numerous health benefits through bioactive compounds (e.g., phytosterols, policosanols) (13, 14). Like other food products, insects undergo structural changes during processing (15, 16). Even though more than 2 billion people worldwide eat insects every day (17), nations, such as Belgium, The Netherlands, and Switzerland, where entomophagy is not common, are regulating insect consumption to ensure its safety.

This paper reviews (a) the impact of insects as food ingredients in the nutritional and techno-functional features, (b) insects as a source of bioactive compounds, and (c) the effect of food processing in nutritive and bioactive components of insects. Furthermore, (d) microbiological safety aspects of consumption and regulations of insects are discussed, along with the importance of consumer perception.

## Food Applications

### Insect-Enhanced Foods

#### Cereal-Based Foods

Cereal-based foods have long been a staple of many cultures around the world, and they are the most important source of nutrients for humans. The most consumed cereals are rice, wheat, and maize (18). Unfortunately, some of them lack important nutrients, for example, maize is a deficient source of Fe, Ca, and Zn, and has only 8% protein. This is why, in countries where micronutrient deficiencies are considered moderate or severe public health problems, cereal meal enrichment with selected vitamins and minerals has been used to improve micronutrient intake and prevent deficiencies (18, 19).

The incorporation of edible insects to enrich cereal products has been already suggested by some authors. Insect flours have been used to replace 5–40% of cereal flour in staple foods or snacks, with the ideal replacement around 10% (Table 1). Wheat bread has been enriched with flour from cinereous cockroaches (*Nauphoeta cinerea*), black soldier fly larvae (*Hermetia illucens*), adult crickets (*Acheta domesticus*), and yellow mealworm (*Tenebrio molitor*) for protein enrichment (10, 20). In dough and bread, *A. domesticus, H. illucens*, and *T. molitor* were used to replace 5% wheat flour. Fat content of insect flours ranged from 27 to 36% dry weight (dw) and protein content from 45 to 57% (dw). The bread-making process was achieved with all the composite flour without significantly affecting dough properties, resulting in bread with acceptable technological quality and an improved nutritional profile. Protein, lipids, and fiber levels have increased an average of 12.7, 246, and 120%, respectively (20). In this way, cinereous cockroaches (63% protein and 23% lipids, dw) replaced 5, 10, and 15% of wheat flour in the bread formulation where the fat content of the insect flour was deducted from the formulation (Table 1). Bread made with cockroach flour has an advantage in terms of fat content, with a high percentage of unsaturated fatty acids rich in omega-6 (ω-6) and omega-9 (ω-9). Bread enriched with 10% cockroach flour presented the best nutritional characteristics (protein increased 49%), without alterations in sensorial quality (low crumb hardness values and a high total quality score) when compared with white and whole wheat bread. Increased cockroach flour content resulted in higher crumb compression values (gluten network formation was altered) in both white and whole-wheat bread. Overall, technical characteristics of composite bread were not altered in a negative way by the addition of cockroach flour (10), although the high-fat content of the insects must be considered in the formulation of the final product.

**Table 1:** Benefits and challenges in the incorporation of insects in food products.

**Food products**	**Insect (replacement %)**	**Technological challenge**	**Benefit**	**References**
Wheat bread	*Hermetia illucens, Acheta domesticus* and *Tenebrio molitor* (5%)	Insect's enriched breads had high fat content incorporated by high fat content in insects. The fat content of the insect flour must be subtracted from the fat in the formulation.	Protein, lipids and fiber levels were increased an average of 12.7, 246, and 120%, respectively.	(20)
	Cinereous cockroach *(Nauphoeta cinerea)* (5–15%)		Large percentage of unsaturated fatty acids rich in ω-6 and ω-9 were found. Protein increased to 49% by 10% wheat flour replacement.	(10)
Maize tortilla	*T. molitor larva* (6.5%)	Tortillas were darker than the control. Consequently, larva was dried at 60°C, temperature in which develops less color; at higher temperatures, the larvae turn a dark brown color.	Protein and fat content increased by 2 and 1%, respectively, as did essential amino acids (phenylalanine, tyrosine, and tryptophan) and polyunsaturated acids (linoleic acid).	(21)
Extruded cereals snacks	Grasshopper (*Sphenarium purpurascens* Ch.) (0–40%)	Increasing insect proportion decreased expansion index; hardness and water absorption index and increased the bulk density and total color difference. Meal proportion was optimized to improve nutritional while maintaining product quality.	A consumer-friendly extruded snack can be made from a combination of nixtamalized maize flour and grasshopper meal.	(22)
	Yellow mealworm larvae (*T. molitor*) (10 and 20%)	Increased barrel temperature and screw speed improved snack microstructure (expansion and pore structure), resulting in acceptable textural qualities.	Protein and fat content increase 35 and 288% respectively. Digestibility of *T. molitor* proteins was improved by 33%.	(17)
Wheat cookies	Palm weevil larvae *(Rhynchophorus phoenicis)* (10–50%)	As the levels of larva substitution increased, the cookies became softer and very dark in color. Meal proportion was optimized to improve nutritional while maintaining product quality.	Cookies containing 10% insect larvae had higher protein (increased 86%), fat (increased 30%) and fiber (increased 642%) content.	(23)
Pork emulsion sausages	Mealworm larvae *(T. molitor)* and silkworm pupae *(Bombyx mori)* (10%)	Fat content increased by 5% and moisture content decreased by −8% with mealworm larvae and silkworm pupae. *A. domesticus*was used (less amount of fat). No significant differences were found in fat content or moisture content of meat emulsions.	In emulsion sausages, the protein content increased by 21%, and almost all minerals were increased (e.g., P, K, Ca, Mg, ZN, Mn)specially Zn that increased 89%, Ca and Mg that double its amount and Cu increased 6 folds; mealworm larvae flour contributed to Fe increases by 1.5 folds.	(24)
	Cricket *(A. domesticus)* (5–10%)		As replacement level increased, P, K, Mg, Zn, and Mn contents of meat emulsion were increased. Compared to regular formulation (control emulsion), insect treatments had higher protein 18–48%.	(11)
Soy meat analog	*Alphitobus diaperinus* (15–50%)	The addition of soy fiber (5–10%) to samples improved cutting strength to levels comparable to chicken breast meat.	Meat analogs with 25–31% of protein content.	(25)
Insect—soy like- fermented sauce	*T. molitor* larvae (60–80%)	Browning increased as fermentation progressed in the insect sauces. Browning of defatted insect sauce increased but then dropped dramatically on day 20. The use of sauces with a 60%ratio resulted in higher amino nitrogen content (0.26–0.32%) than sauces with a 80% ratio, indicating more efficient protein degradation.	During fermentation, essential and non-essential amino acids, as well as amino acid derivatives, increased by 1.5–two times.	(26)
Honey spread	Soldier termites (*Syntermes soldiers*) (8%)	Honey spread with soldier termite flour processed by pan-frying rather than boiling water at 100°C rather than 80 or 90°C had good nutritional and sensory qualities.	Protein increased from 0.4 to 5.5% and Fe and Zn solubility increased to 42.8 and 27.1%, respectively with contents of 3.80 mg/100 g and 1.75 mg/g	(9)
Insect tea	Produced using insect feces fed from tea leaves [*Aglossa dimidiata Haworth, Hydrillodes morose Butler*, and *Nodaria niphona* (Butler)].	Low production rate and long production time (1 year).	Higher levels of human essential amino acids such as valine (3 folds), threonine (2.45 folds), and phenylalanine (2.35 folds).	(27)
Wheat based feed	*H. illucens* (25%)	*H. illucens* prepupae was not extrudable, by supplementing it with fat (from 3.9 to 4.6%) an acceptable level for extrusion was achieved. Therefore, larvae (with higher fat content than prepupae) was used and the best extrusion performance was obtained.	Extrusion process increased *in vitro* organic matter digestibility by 16.8% compared to unextruded control.	(12)
Fishmeal based fish feed	*H. illucens* and *A. domesticus* (0–75%)	As the level of fish meal substitution increased, Fe and Na levels decreased significantly. Mg content increased with increasing substitution level of *H. illucens* meal but decreased with increasing substitution level of *A. domesticus*meal. As a result, use *H. illucens* and optimize to prevent Fe levels from dropping to suboptimal levels.	Most minerals were less leached by the diets than by the control diets. P and K levels increased as the level of fish meal substitution increased.	(28)

The main problem with baked products is the change in color in comparison to control (without insects), specifically in crumbs, which shows different brownish intensities, but this issue does not interfere with dough fermentation, resulting in bread with acceptable volume and open crumbs, and it does not adversely affect the technical characteristics. Furthermore, sensorial analysis was satisfactory with a positive attitude of purchase (20). Besides research, several commercial initiatives have sprung around the use of insects as an alternative to traditional Western ingredients in bakery products. For example, Fazer—a Finnish bakery—started producing and selling bread with cricket flour in 11 of its stores, with plans to expand throughout all its 47 stores within a year (29).

Another cereal-based staple product, a maize tortilla, has been enriched by replacing nixtamalized maize flour with 6.5% of yellow mealworm larva flour (*T. molitor*) (21). Maize tortillas protein, fat, amount of essential amino acids, and polyunsaturated acids increased. Tortillas were darker than the control; consequently, a larva was dried to avoid darker color (Table 1). The supplemented tortilla had a pleasant taste, texture, and color. Additionally, cricket tortilla chips have been used in consumer preference studies, using commercial cricket flour (Six Foods LLC) and deep-fried cricket bits (30). The enrichment of wheat and maize flour with different insects to increase the proportions of protein and fat is feasible and convenient to prevent nutritional deficiencies in populations that have difficult access to protein-rich food.

In contrast, snacks have evolved into an important component of the eating habits of the global population. Exo is a U.S.-based company producing commercially available cricket flour snack protein bars, which was recently acquired by Hult-prize winner Aspire Food Group, a producer of cricket and weevil flour (31). The most popular snacks are made primarily of maize and wheat. Recently, both maize and wheat ready-to-eat extruded snacks enriched with insects have been developed (17, 22). Nixtamalized maize flour (10.5% protein) enriched with grasshopper meal (*Sphenarium purpurascens* Ch.) (52.7% protein) was used to formulate a snack, using a single screw extruder (22). Increasing the proportion of grasshopper meal (0–40%) decreased the expansion index, hardness, and water absorption indexes, while increasing bulk density and total color difference (Table 1). Treatments with a lower proportion of grasshopper meals had higher overall acceptability. A consumer-acceptable extruded snack was obtained from a blend of 92% nixtamalized maize flour and 8% grasshopper meal without affecting the physicochemical properties or acceptance of the snack. Likewise, yellow mealworm larvae (*T. molitor*) flour was used to replace 10 and 20% of wheat flour in extruded cereal snacks (Table 1). Snacks enriched with 10% mealworm powder had a change in protein from 11.8 to 15.9% and lipid composition from 0.9 to 3.5%. The use of a high barrel temperature and screw speed at 10% enrichment significantly improved expansion and pore structure, resulting in acceptable textural qualities. Snacks had poor expansion properties at 20% substitution due to the presence of larvae fat (5.4%), which reduced viscosity and mechanical energy. Mechanical forces generated during extrusion increased the digestibility of *T. molitor* proteins by 33% in those enriched at 10% mealworms (17). Similarly, snack cookies using wheat flour have been supplemented with palm weevil larvae flour (10–50%). Cookies containing 10% palm weevil larvae (*Rhynchophorus phoenicis*) flour had higher protein content (increased by 86%), fat (increased by 30%), and fiber (increased by 642%). There was no significant difference in the taste, appearance, texture, and overall acceptability of cookies made from 100% wheat flour and 10% palm weevil larvae substitution (23).

In addition, insects are also rich in dietary relevant minerals, increasing their content on processed foods, for example, deep-fried wheat-maize snack buns enriched with 5% of *R. phoenicis* larvae increased their protein, Ca, Mg, and Zn content by 20, 12, 32, and 88%, respectively. Sensory evaluation showed no significant differences in an aroma, flavor, taste, texture, and general acceptability against 100% wheat buns. The values of the dough swelling index, bulk density, and oil absorption capacity did not differ significantly (32, 33). Likewise, edible winged termites (*Macrotermes subhylanus*) have been used to substitute wheat flour by 5%. There was no significant difference in bun height and size between the buns with 0% and 5% termite concentrations, nor in scores for bun texture, aroma, taste, or overall consumer preference. The 5% substitution resulted in a significant increase (*p* ≤ 0.05) in protein (47.5%), iron (50%), riboflavin (53%), zinc (16%), and niacin (23%) contents (34). In this way, snacks are an ideal vehicle to introduce insects as ingredients to conventional preparations such as wheat and maize, increasing the nutrimental composition of these daily high-consumed products. As the food industry looks for alternative ingredients, cereal products can be processed with the inclusion of insect flour to enhance the nutritional quality of the product without affecting product quality characteristics nor compromising the technological properties of the products.

#### Animal-Derived Protein Foods

Insects have caught the interest of Western societies in recent years, particularly as an alternative source of animal protein. The low environmental cost of insect protein is one of its main advantages over other sources while offering technological advantages such as gel-forming ability, emulsion capacity, and water/oil absorption ability (35). Insect proteins have recently been used to partially replace meat in processed meat products while maintaining technological and nutritional properties (Table 1). For example, mealworm larvae (*T. molitor*) and silkworm pupae (*Bombyx mori*) flour were used to replace 10% of the lean pork in emulsion sausages (24). In comparison with the standard formulation (control sausage), insect treatments had higher protein and fat content (21 and 5%, respectively) and lower moisture (−8%). Protein solubility slightly decreased from 17.36 mg/g to 16.82–16.93 mg/g, whereas sausage pH increased from 6.04 to 6.32–6.37. All emulsion sausages containing insect flour had an increased cooking yield (2%) and firmer texture than control sausage due to the decreased moisture. Moreover, the addition of mealworm larvae insect flour led to increases in cohesiveness and chewiness. The addition of insect flour increased the amounts of almost all minerals in emulsion sausages significantly (e.g., K, P, Ca, Zn, Mg, and Mn), especially Zn that increased 89%, Ca and Mg that doubled their amount, and Cu, which increased 6-fold; mealworm larvae flour also contributed to a 1.5-fold increase in Fe (24). Subsequently, cricket (*A. domesticus*) flour was added to meat emulsions in six treatments: lean meat (5%), fat (5%), lean meat (2.5%) + fat (2.5%), lean meat (10%), fat (10%), and lean meat (5%) + fat (5%) (11). As the replacement level increased, P, K, Mg, Zn, and Mn contents of meat emulsion were increased. Compared with the regular formulation (control emulsion), insect treatments had 18–48% higher protein content, and no significant differences were found in fat content or moisture. Emulsions pH, cooking yield and texture springiness, and cohesiveness had no significant differences among formulations, with only higher texture hardness in insect emulsions (11). This study represents a clear and real approximation of obtaining a product elaborated with an alternative source of protein with similar content of fat and moisture, enhancing protein and minerals content.

In the same way, high-moisture extrusion was used to generate meat analog products, using a twin-screw extruder with *Alphitobus diaperinus* flour (60% protein dw). Extruding insect concentrate (ranging from 15 to 50% dry matter), soy concentrate, and water-produced fibrous meat analogs with meat-like hardness and protein composition. A mixture of 40% *A. diaperinus* and 60% soy dry matter produced the best results (most similar to standard soy-based samples). The inclusion of soy fiber (5 or 10%) in samples with 40% water content improved cutting strength, resulting in parameters similar to chicken breast meat. The resulting meat analogs had a protein content between 25 and 31%. Increasing the concentration of insect protein powder (at 170°C and 45% humidity) reduced the cutting strength in the samples. It has also been reported that increasing the insect content in the formulation resulted in lower texture lightness. Increasing water content in the samples resulted in increased protein solubility (25). Therefore, a combination of a vegetal and insect protein source is proposed to originate a meat analog.

Nowadays, commercially available substitutes of animal protein include C-fu Foods, which is a company that produces a tofu-like “burger,” made entirely with mealworm protein, and containing as much protein (pound-for-pound) as an egg. This burger is also high in omega-3 (ω-3) and ω-6 fatty acids. Another example of insects in animal-derived products includes casumarzu cheese, a sheep cheese found only in Sardinia, with a ripening period during which the cheese is allowed to be infested with *Phiophilacasei* eggs. Larvae derived from eggs feed on cheese, promoting chemical modification and conferring the typical organoleptic properties (36). In the future, insect proteins and fats could be used to assess the technical feasibility of an insect-derived cheese or an egg substitute.

#### Animal Feed

The livestock sector is under increasing strain to meet the rising demand for high-value animal protein, implying several feed supply problems, particularly feed protein supply issues (12). One solution to the expected scarcity of dietary protein for animal feeds may be to replace current protein resources with novel or alternative sources. In this context, insects have been proposed as a possible source of animal feed protein.

*Hermetia illucens* larvae and prepupae replaced 25% of wheat in experimental extruded feeds (12). A co rotating, conical twin-screw mini extruder was used to extrude five mixtures, four with prepupae + four levels of added fat (0–22.5%) and one with larvae without added fat. Blends were homogeneous for moisture (24%) and crude protein [11% wet basis (wb)]. The two lowest fat mixtures (3.1–3.9%) were considered not extrudable (net torque value >100 Ncm). The net torque value was reduced significantly (up to four times) to an acceptable level for extrusion by increasing the fat content from 3.9 to 4.6% wb. The extrusion process increased *in vitro* organic matter digestibility by 16.8% compared with unextruded controls (Table 1). The highest fat mixtures, prepupae: wheat, 25:75 (high oil added) (5.3% fat) and larvae: wheat, 25:75 (no oil added), produced the best comparable extrusion performance (4.6% fat).

Moreover, *H. illucens* larvae have also been used to study the effect on minerals content of substituting a fish meal with insect meal in extruded fish feeds (37). In that study, Black soldier fly larvae meal (*H. illucens*) and adult cricket (*A. domesticus*) meal substituted fish meal on four levels (0, 25, 50, and 75%) (28). The results revealed a significant increase in K and P levels as the level of fish meal substitution increased from 0 to 75%. Nevertheless, as the level of fish meal substitution increased, Fe and sodium (Na) levels decreased significantly (Table 1). Mg content increased with an increasing substitution level of *H. illucens* meal but decreased with an increasing substitution level of *A. domesticus* meal. Cu, Zn, and Mn concentrations were unaffected by fish meal substitution levels. Diets with no substitution had a higher leaching effect for most minerals than diets with 75% insect meals substituted. In fish feeds, insect meal can be used to replace up to 75% of the fish meal while supplying adequate quantities of minerals. Feeding fish trials revealed that *T. molitor* meal could be used in place of fish meal in feeds without compromising growth performance as no detrimental effects on growth performance were found (37, 38). Additionally, some filet quality parameters, such as water-holding capacity and texture characteristics, showed no significant differences (hardness, cohesiveness, resilience, gumminess, and adhesiveness).

#### Other Insect-Based Food Products

Given its high-protein content, insects could be used as an alternative raw material for high-protein quality foods in the future. In order for insect proteins to be used as edible food (e.g., sauces, beverages, spreads, and protein powders to gain muscles), processing technologies must be developed to make the insect industry economically competitive (3).

Honey enriched with soldier termites (*Syntermes soldiers*) flour was used to develop a nutritive spread (9). Spreads enriched with 8% soldier termite flour and pan fried at 100°C had good nutritional and sensory properties (Table 1). Product color and spreadability were rated high. Protein increased from 0.4% of honey to 5.5%, and Zn and Fe solubility increased to 27.1 and 42.8%, respectively, with contents of 3.80 mg/100 g and 1.75 mg/g, respectively. In the same way, insect tea, which is produced using insect feces fed from tea leaves, is a traditional drink that ethnic minorities in the southwest of China have long consumed. Compared with tea leaves, insect tea had a significantly higher level of total amino acids. The insect tea also had higher levels of essential amino acids for humans such as threonine (2.4-fold), valine (3-fold), and phenylalanine (2.3-fold). Isoleucine content in insect tea was 0.077 mg/100 g and was not detected in tea leaves. When compared with raw tea, insect tea had higher levels of glutamate, arginine, and proline (27).

Likewise, using *T. molitor* larvae and the soy sauce fermentation process, a liquid-fermented seasoning was created (Table 1). Six sauce samples, including *T. molitor* larvae, defatted larvae, and soybeans at two different ratios (60 or 80% protein source) were fermented for 20 days. Browning increased as fermentation progressed in the soy and raw insect sauces. The browning of defatted insect sauce increased but then decreased dramatically on day 20 (Table 1). There was no significant difference in pH between samples. The 60% ratio sauces showed greater amino nitrogen content (0.26–0.32% on day 20) than did the 80% ratio sauces, indicating more effective protein deterioration. The concentrations of essential and non-essential amino acids, as well as amino acid derivatives, increased by 1.5–2 times during the fermentation process. The raw 60% insect sauce contained the most total free amino acids (510.42 mg/kg). A soy analog insect sauce was technically feasible (26). Defatted insects can be used as raw materials to produce high-protein foods, and the fats obtained from the defatting process could be used to generate fat-based food products, such as mayonnaise.

Furthermore, insects have also been used in world-renowned restaurants. Pujol, a famous restaurant in Mexico City, offers mayonnaise flavored with a Chicatana ant (*Atta Mexicana*), traditionally used to prepare sauces in the Oaxaca region. As well, many local products using dried insects as ingredients can be found, such as salt, sauces, mayonnaise, chili powders, and others. Moreover, for the prototyping process, the powder was combined with other food products such as chocolate, icing butter, cream cheese, and spices, such as cinnamon, ginger, and dried chilies to obtain 3D print insect-enriched food (4). 3D printing is a novel processing technology to enhance insect-based food appearance and to improve acceptability. Different types of food products can be enriched with insect-derived ingredients in order to increase their nutrimental benefits.

### Insects: Source of Bioactive Compounds

In addition to nutritional benefits, some studies show that insects provide numerous health benefits *via* bioactive compounds. Bioactive compounds are found in both plant and animal products. Bioactive substances found naturally in food provide health benefits in addition to the basic nutritional value of the products. Bioactive compounds are food ingredients that have an effect on the physiological or cellular activities of the animals or humans who consume them (39). The bioactivity of the compounds can range from being an antioxidant compound, cardioprotective, antimicrobial, antifungal, anti-inflammatory, and antitumor, among others (16, 40–45). In Table 2, different bioactive compounds that have been identified in insects are presented.

**Table 2:** Bioactive compounds from insects and their biological activity.

**Insect**	**Bioactive compound**	**Biological activity**	**References**
Cricket *(Acheta domesticus)*	Aqueous extract	In the FRAP and ORAC assays, the antioxidant capacity was 9,285 mM ascorbic acid equivalents/g dw and 296 μM trolox equivalents (TE)/g dw, respectively.	(40)
*Holotrichia parallela* Motschulsky	Aqueous and ethanolic extracts	Metal-chelating activity and inhibition of lipid peroxidation. Metal-chelating activity and antioxidant in the scavenging of DPPH radicals.	(46)
Lucanid beetle (*Serrognathus platymelus castanicolor* Motschulsky)	Methanolic extracts	Scavenging effects on ABTS^•+^. Scavening effects followed the order pupae, adult, and larvae having pupae stage extracts enhanced antioxidant activity.	(47)
Bush cricket (*Brachytrupes orientalis)*	Aqueous extract	In high glucose treated cells, inhibit ROS and increase glucose metabolism by upregulating AMPK phosphorylation and GLUT4 expression using a C2C12 mouse myoblast cell model.	(48)
*Tenebrio molitor* larvae	Extract	In mice, it regulates food intake and body weight by inhibiting the expression of the orexigenic neuropeptides neuropeptide Y and agouti-related protein via hypothalamic mTOR and MAPK signaling pathways.	(49)
Cricket *(Gryllodes sigillatus)*	Protein hydrolysate	Glycemic control-inhibit dipeptidyl peptidase IV (DPP-IV), which is involved in the regulation of insulin secretion and glycemia.	(50, 51)
*Amphiacusta annulipes*	Protein hydrolysate	Antiradical activity against DPPH, Fe^2+^ chelation ability and reducing power.	(16)
*Zophobas morio*	Protein hydrolysate	Antiradical activity against ABTS^+^	
*Locusta migratoria*	Protein hydrolysate	Ability to chelate Cu^2+^	
Locust *(Schistocerca gregaria)*	Protein hydrolysate	Anti-inflammatory activities—inhibits lipoxygenase	(52)
Silkworm Pupae *(Bombyx mori)*	Protein hydrolysate	SGC-7901 inhibits proliferation and induces abnormal morphologic features in human gastric cancer cells in a dose- and time-dependent manner, as well as inducing mitochondria-dependent apoptosis and cell-cycle arrest in S phase.	(53)
*Sarcophaga carnaria*	Fatty acids (short chain, unsaturated and polyunsaturated)	Antifungal and antimicrobial activity	(54)
*Pyrrhocoris apterus*	Phospholipids	Lowering liver lipid levels has the potential to interfere with sterol absorption in the intestinal lumen. They stimulate bile acid and cholesterol secretion and have the ability to raise plasma HDL levels.	(55, 56)
*Ericerus pela*	Policosanols	Reduce lipid levels in the blood and platelet aggregation. Reduce cholesterol by inhibiting endogenous cholesterol biosynthesis through the activity of the enzyme 3-hydroxy-3-methyl-glutaryl CoA (HMG-CoA) reductase.	(14, 57–60)
Linden bug *(Pyrrhocoris apterus)*	Phytosterols (β -sitosterol and campesterol)	Phytosterols are hypocholesterolemic and prevent cardiovascular disease by inhibiting lipid peroxidation in human low-density lipoproteins.	(19, 61–63)
*T. molitor* larvae, *Oedaleus decorus*, and *Hermetia illucens*	Dietetic fiber	Reduced risk of coronary heart disease, stroke, hypertension, diabetes, obesity, and some gastrointestinal diseases. Lowers blood pressure and serum cholesterol levels, improves gastrointestinal function, and protects against constipation, diverticulosis, colon cancer and hemorrhoids.	(45, 64–67)
*H. illucens, Calliptamus barbarous*, Mayflies (order *Ephemeroptera*)	Chitin and chitosan	Chitin fragments of <40 μm have anti-inflammatory properties, inducing the release of IL-10 and regulating the intensity and chronicity of local inflammation. Reduces plasma cholesterol and triglycerides by binding dietary lipids. Lipid absorption in the intestine is reduced.	(43, 45, 64, 65, 68)
Mayflies (order *Ephemeroptera*)	Chitosan	Antitumor activity in HeLa cells (cervical cancer), and inhibitory activity on A549 (lung) and WiDr (colon) cancer cells	(41, 45)
*Julus terrestris, C. barbarus* and *O. decorus*	Chitosan	Antimicrobial activities for common human pathogen (e.g., *Staphylococcus aureus, E. aerogenes, S. epidermidis wt, B. subtilis, S. typhimurium, C. albicans and P. aeruginosa*) and yeast (*Candida albicans*).	(41, 65)

Antioxidant agents can slow oxidation in foods, quench metabolically generated reactive oxygen species (ROS), protect against ROS damage, and lower the risk of chronic disease in humans (46). One of the most studied edible insects is *A. domesticus*. Antioxidant capacity from an aqueous extract of *A. domesticus* flour has been measured by FRAP (ferric reducing ability, based in single electron transfer) and ORAC (oxygen radical absorbance capacity, based in hydrogen atom transfer) assays. *A. domesticus* extracts antioxidant capacity (Table 2) was 9,285-mM ascorbic acid equivalents/g dw and 296-μm Trolox equivalents (TE)/g dw in the FRAP and ORAC assay, respectively (40); similar to blueberries (240 μm TE/g dw) (69), widely recognized as antioxidants. Likewise, extracts of the adult black chafer beetle *Holotrichia parallela* Motschulsky (eaten in China and Southeast Asia) with antioxidant activity have been reported (46). Metal-chelating activity and inhibition of lipid peroxidation were demonstrated by ethanolic extract. Water extract outperformed butylated hydroxytoluene (BHT) antioxidant (EC_50_: 3.51 mg/ml) in the scavenging of 2,2-diphenyl-1-picrylhydrazyl (DPPH) radicals (EC_50_: 1.45 mg/ml) (Table 2). Catechin (7.66 ± 0.05 mg/g extract) was identified in the ethanolic extract, that it could be derived from the cuticles of the insect species or from the plants that the insects consume.

Although beetles are eaten in the adult stage, the lucanid beetle (*Serrognathus platymelus castanicolor* Motschulsky) antioxidant activity has been studied in different growth stages (47). It was found that the pupae stage methanolic extracts had higher antioxidant activity than adults or larvae. The scavenging effects of various extracts on the ABTS^•+^ followed the order pupae, adult, and larvae (Table 2) and were 79.6, 53.3, and 22.9%, respectively (47). The same order was followed in the quenching effect on the singlet oxygen: pupae, adult, and larvae with values of 95.3, 50.8, and 14.5%, respectively (47). Insects could be used as a nutraceutical to treat oxidative stress-related diseases, as well as natural antioxidant additives in the food industry.

Bush crickets *Brachytrupes orientalis* is a common edible insect species consumed by various tribes in North East India. In high-glucose-treated cells, using a C2C12 mouse myoblast cell model, aqueous extract of *B. orientalis* supplementation can inhibit ROS and increase glucose metabolism via upregulation of AMP-activated protein kinase (AMPK) phosphorylation and glucose transporter type 4 (GLUT4) expression (Table 2) (48). Similarly, *Allomyrina dichotoma* larvae administered to mice in a high-fat diet prevent an increase in body weight [22.4% lower compared with mice fed only high-fat diet (HFD)] and organ weight, particularly epididymal adipose tissue weight (70). Moreover, serum triglyceride and leptin were reduced in mice fed with HFD plus a 3,000-mg/kg days of *A. dichotoma* larvae (70). Likewise, *T. molitor* larvae extract administrated intracerebroventricular to mice in HFD regulates food intake and body weight by suppressing the modulation of hypothalamic mammalian Target of Rapamycin (mTOR) and mitogen-activated protein kinase (MAPK), signaling pathways to increase the expression of orexigenic neuropeptide Y and agouti-related protein (Table 2) (49).

Insect tea, produced using insect feces, is a traditional ethnic drink in southwest China. Insect larvae feed on leaves, excreting insect feces particles. Insect tea was produced from Kuding tea leaves. Insect tea polyphenol-rich extract was administered to Kunming mice (200 mg/kg). Mice showed higher superoxide dismutase, glutathione peroxidase, and glutathione activities while having lower levels of nitric oxide, and malonaldehyde activities than Kuding tea polyphenol-rich extract (71). In addition, 100 mg/kg of insect tea reduces the acute gastric injury caused by hydrochloric acid and ethanol in mice (27). With insect tea treatment, the level of inhibition of gastric ulcer area was 72%. Treatment with insect tea significantly increased vasoactive intestinal peptide and somatostatin, and significantly decreased motilin, and endothelin levels in the serum; thus, insect tea can be used as a functional food. Insects have been underutilized as potential sources of natural antioxidants and a source of bioactive compounds. They have numerous health benefits and can be developed into nutritious foods.

#### Bioactive Peptides

The protein content of insects is extremely high, reaching around 60% in many species; for example, locusts, grasshoppers, and crickets can have up to 77% protein content (dw) (17). This is one of the reasons why there has been interest in them as food in recent years. Its proteins, apart from giving nutrients and essential amino acids to those who consume them, also provide bioactive peptides. Recently, reviews have been published on bioactive peptides obtained from edible insects (72); but given their importance, the most recent research is reviewed below.

Protein hydrolysates from *Gryllodes sigillatus* (crickets) can inhibit dipeptidyl peptidase IV (DPP-IV), which is involved in the regulation of insulin secretion and glycemia (Table 2), conferring *G. sigillatus* protein hydrolysates, a potential role in glycemic management (50, 51). Protein hydrolysates inhibited an angiotensin-converting enzyme; which helps to relax blood vessels and treat a variety of conditions, such as scleroderma, high blood pressure, and migraines (50).

Among these applications, the medicinal properties of edible insects are primarily derived from the extraction and development of antibody peptides. Current research and development status of antimicrobial peptides include Intrabiotics Pharmaceuticals with a peptide IB-367 (Iseganan) Protegrin analog to treat mucositis in phase III, Entomed SA with peptide Heliomycin with antifungal applications in the preclinical stage, and Demegen with P-113 histatin analog peptide with gingivitis application in phase II (73).

Edible insects are a good source of peptides that have antiradical activity as well as the ability to chelate metal ions and inhibit reducing power (50, 52). Edible insects have higher antioxidant activity after simulated gastric digestion than some protein hydrolysates derived from plants or other animal products. *Amphiacusta annulipes* had the highest antiradical activity against DPPH (19.1 g/ml), while *Zophobas morio* had the highest antiradical activity (4.6 g/ml) against ABTS [2,2'-azino-bis(3-ethylbenzothiazoline-6-sulfonic acid)], a chemical compound used to determine antioxidant capacity. *A. annulipes* peptides also had the highest Fe^2+^ chelation ability (58.8%) and reducing power (0.652) (Table 2). *Locusta migratoria* had the greatest ability to chelate Cu^2+^ (86.1%). The locust had the highest peptide concentrations before and after digestion (3.13 and 5.88 mg/ml, respectively), and the degree of hydrolysis (DH) was 36.3% (52). Consumption of edible insects may be beneficial to the health of human body.

Subsequently, it was discovered that edible insects are a good source of peptides with anti-inflammatory properties. *Schistocerca gregaria* (locust) protein hydrolysate inhibits lipoxygenase [half-maximal inhibitory concentration (IC_50_) value 0.65 mg/ml] (Table 2). Generally, the best anti-inflammatory activity [inhibitory activity of lypoxigenase (LOX) and cyclooxygenase (COX)] was found in the peptides and hydrolysates obtained after digestion of the insect proteins rather than in raw insects (16).

Silkworm pupae (*B. mori*) are widely consumed in China. In a dose- and time-dependent manner, protein hydrolysates from *B. mori* can specifically inhibit cancer cell proliferation and induce abnormal morphologic features in human gastric cancer cells (SGC-7901) (Table 2). Silkworm pupa protein hydrolysates cause a mitochondria-dependent apoptosis and cause the cell cycle to stop in the synthesis (S) phase. Peptides extracted from silkworm pupae had the potential to be used for the therapeutic management of gastric cancer in the future (53). Insect proteins are not only valuable for their nutritional value but for their proven therapeutic effect.

#### Lipids

Lipids, after proteins, are the most important compounds of edible insects. The insect *Encosternum delegorguei* is consumed as human food in Zimbabwe and has a high fat content of 50.6% edible weight (3). Likewise, eaten in Zimbabwe, the fat content of the mopane caterpillar (*Gonimbrasia belina*) is 16–20%, with essential fatty acids accounting for 40% (3). Essential fatty acids, such as linoleic acid, are particularly abundant in the African aboveground hill termite species, *Macrotermes subhyalinus* (43%), and *Macrotermes bellicosus* (34%) (3). There is increasing awareness of the role of polyunsaturated acids (PUFA) as food supplements in the human diet and in the prevention of different diseases [e.g., linolenic fatty acids (ω-3) exert healthy effects in the care of cardiovascular diseases] (74). Insects may play a significant role in supplying these essential fatty acids; the composition of unsaturated ω-3 and ω-6 fatty acids in mealworms is comparable with that in fish (3). It has also been found that lipids from *Blaptica dubia, T. molitor, A. domesticus*, and *A. diaperinus* contain ω-3 and ω-6 fatty acids (75).

Insect feeding can change the nutritive values of insect fats. The addition of flaxseed in *Galleria mellonella* feed increases ω-3 fatty acid content from 2 to 14.8% and ω-6 fatty acid values from 9.9 to 12.6% in *G. mellonella* larvae. Larvae fed showed a ω-6/ ω-3 ratio optimal for human health and total PUFA incremented from 9.5 to 25.5% (74). Moreover, the use of insect fatty acid desaturases modifying saturated fats to PUFA could be foreseen (76).

Apart from containing essential fatty acids and PUFA, and exert healthy effects, it has been demonstrated that fatty acids, as components of insect cuticular lipids, play an important role in fungal infection resistance. Fatty acids from *Sarcophaga carnaria* were tested vs. entomopathogenic fungi: *Paecilomyces fumosoroseus, Paecilomyces lilacinus, Lecanicillium lecanii, Beauveria bassiana*, and *Metarhizium anisopliae*. The antimicrobial activity of fatty acids varied, depending on the length of the chain and the presence of unsaturated bonds. Short chain and unsaturated fatty acids (6:0, 11:0, and 13:0) have significantly higher antifungal activity, whereas PUFA inhibit fungal growth more effectively than unsaturated long chain fatty acids (Table 2) (54).

Not only insect lipids are constituted of triacylglycerols, but also phospholipids, sterols, waxes, glycolipids, among others. Triacylglycerols account for approximately 80% of the lipid content. Phospholipids, the second most important lipid class, play an important role in membrane cell structure. The phospholipid (PL) content of crude fat is typically <20% (75). Like triglycerides, the backbone of phosphoglycerides is glycerol, but only the primary and secondary glycerol alcohol residues are esterified to fatty acids; the third site is esterified to a phosphate group, which is then linked to an inositol (PI), choline (PC), ethanolamine (PE), glycerol head (PG), or serine (PS) (77). *Pyrrhocoris apterus* analyses revealed that PLs with the PC and PE head groups accounted for more than 80% of total PLs in *P. apterus*, while the less-abundant PLs had the sphingomyelin (SM), PI, PS, and cardiolipin (CL) head groups (56). Phospholipids are beneficial to human health. The majority of the studies found that dietary supplementation has a positive impact on a variety of diseases, including coronary heart disease, inflammation, and cancer (78). Moreover, phospholipids lower liver lipid levels and can inhibit sterol absorption in the intestinal lumen. Phospholipids increase plasma high-density lipoprotein (HDL) levels by stimulating bile acid and cholesterol secretion (55). Phospholipid isolates have the potential to be used as hepatoprotective nutraceuticals. Predominantly, phosphatidyl choline or lecithin aids in the proper functioning of the liver and the transport of lipids and its deficiency is linked to an increased risk of hepatic cancer (19). Lecithin and choline reduce the risk of cardiovascular disease and improve brain and mental development in both fetuses and infants, and their chronic deficiency may be linked to Alzheimer's disease. Choline, which is released from lecithin during metabolism, is one of the most important neurotransmitters and is commonly supplemented in geriatrics to maintain proper brain function (19).

Policosanols are a combination of long-chained aliphatic primary alcohols, primarily docosanol (22:0), hexacosanol (26:0), octacosanol (28:0), triacontanol (30:0), and dotriacontanol (32:0) (79). Their importance in human health stems from their demonstrated efficacy in physiological activities such as lowering serum lipid levels and platelet aggregation (57), lowering total blood and plasma cholesterol [*via* enzyme 3-hydroxy-3-methyl- glutaryl CoA (HMG-CoA) reductase activity (14, 58)], low-density lipoprotein (LDL)-cholesterol and insulin resistance (19, 80), and improving mood state (81) (Table 2).

Chinese Insect Wax is a natural white wax secreted by male *Ericerus pela larvae*. The main chemical components of the wax are esters formed by monoacids such as octacosanoic acid, hexacosanoic acid, tetracosanoic acid, triacosanoic acid, and monohydric alcohols such as octacosanol, hexacosanol, tetracosanol, triacosanol, among others, accounting for 93–95% of the total esters amount (59). In recent years, insect wax has emerged as an important material for the production of policosanols with high yields (723.84 mg/g) and median lethal dose (LD_50_) values for both insect wax and policosanol exceeding 5 g/kg (60), making them potential candidates as supplements in foods.

Phytosterols (C28 or C29 sterols and stanols) are natural components of plant membranes. Insects are unable to synthesize sterols from isoprenoid precursors, so they must be obtained from dietary sources (13). Phytosterols compete for cholesterol absorption and prevent from cardiovascular diseases. Sterols (2 g) inhibit the absorption of cholesterol from the small intestine, lowering total blood cholesterol by 5–20% and decrease oxidized LDL by 10–15% and apolipoprotein B (19, 61, 62). Phytosterols inhibit several pro-inflammatory mediators through the inhibition of the nuclear factor kappa-light-chain enhancer of the activated B cells (NF-kB) pathway (82).

In linden bug fat, *P. apterus*, β-sitosterol, and campesterol (Table 2); two phytosterols derived from plant food; and two isomers of tocopherol (vitamin E) were found (63). Seasonal patterns of sterol and tocopherol concentrations were discovered with a minimum in reproductively active insects in the summer and a maximum in diapausing insects in the winter. In addition, lipids were high in campesterol and β-sitosterol, two phytosterols derived from their food (seeds) (63).

An African desert locust could be used as an unusual source of dietary and therapeutic sterols. Thirty-four sterols were identified in *S. gregaria* lipids [e.g., desmosterol, 7-dehydrocholesterol, (3β, 5α) cholesta-8, fucosterol]. Three sterol derivatives were found in the gut of the locust after consuming a plant-based diet but were not detected in the diet. The desert locust ingests phytosterols and amplifies and metabolizes them into derivatives with potential health benefits (13). Insect lipids contain fat-soluble nutrients as well as bioactive molecules that can help with diseases like coronary heart disease, cancer, and inflammation. Bioactive-derived insect lipids should be used to design foods for improved health.

#### Chitin

Insects contain significant amounts of fiber. Chitin, an insoluble fiber derived from the exoskeleton, is the most common type of fiber in insects (3). Chitin is a linear polysaccharide, consisting of N-acetyl-D-glucosamine residues and accounts for about 10% of the whole dried insect (5, 44). Mayflies (order *Ephemeroptera*) bodies had 10.2% chitin content, *T. molitor* larvae, and *Oedaleus decorus* grasshoppers, 16%, and *H. illucens* and *Calliptamus barbarus* grasshoppers, 20% (45, 64, 65). Chitin content varies between edible insect forms, as seen in *Dendrolimus houi* Lajonquiere pupa (7.4%) and adults (17.8%) (83).

Chitin acts like a dietetic fiber, even though is partially digestible by humans by chitinase [acidic mammalian chitinase (AMCase)] found in gastric juices (3, 68). The intake of dietary fiber provides many health benefits. People who consume a lot of fiber appear to have a lower risk of developing coronary heart disease, hypertension, stroke, obesity, diabetes, and gastrointestinal diseases. Increased fiber consumption lowers blood pressure and serum cholesterol levels, improves gastrointestinal function, and protects against constipation, diverticulosis, hemorrhoids, and colon cancer (66, 67). Chitin and chitosan can bind dietary lipids, lowering plasma cholesterol and triglycerides. As a result, lipid absorption in the intestine is reduced (83).

Chitin and chitosan (produced by deacetylation of chitin) have sparked the interest of researchers due to their biological activity, which includes immune-boosting properties, antimicrobial and antifungal effects, anti-inflammatory effects, and antitumor and antioxidant activity (41, 43–45, 65, 84). For example, chitin fragments of <40 μm have anti-inflammatory properties, inducing the release of interleukin (IL)-10 and regulating the chronicity and intensity of local inflammation (Table 2) (43). *Grasshoppers O. decorus* and *C. barbarus* chitin antioxidant capacities were evaluated, using the free radical scavenging activity (DPPH). The IC_50_ values for *C. barbarus* and *O. decorus* chitins were 10.68 and 10.91 mg/ml, respectively, which were higher than the value for BHT:0.04 mg/ml (65). Moreover, chitosan antitumor activity tests were conducted in HeLa cells (cervical cancer), low cytotoxicity, and high antiproliferative activity was observed at 500 μg/ml and Mayfly (order *Ephemeroptera*) chitosans (250 μg/ml) exhibited significant inhibitory activity on lung and colon cancer cells (Table 2) (41, 45).

The antimicrobial activity of *Julus terrestris* chitosan was tested on 12 microorganisms, with the most inhibition (15.6 mm) for the common human pathogen *Staphylococcus aureus* and lowest activity against *Klebsiella pneumoniae* (Table 2); other microorganisms in which a chitosan has antimicrobial activities include *Enterobacter aerogenes, Staphylococcus epidermidis wt, Bacillus subtilis, Salmonella typhimurium, Candida albicans*, and *Pseudomonas aeruginosa* (41). Likewise, a chitosan obtained from *C. barbarus* and *O. decorus* exhibited potent antimicrobial activity against pathogenic microorganisms: *B. subtilis, Listeria monocytogenes, Yersinia enterocolitica*, and *Salmonella enteritidis*, and one type of yeast (*C. albicans*) (65). Likewise, antifungal chitosans were active (25 mg/ml) on three tested fungi (*Alternaria solani, Botrytis cinerea*, and *Aspergillus niger*) (44).

Because of a chitosan's excellent properties, such as adsorption, film formation, and antimicrobial properties, the chitin is used in food preservation (44). The chitin has an anti-inflammatory effect on the immune system. By feeding insects to poultry, the use of antibiotics of the industry, which may lead to human infection with drug-resistant bacterial strains, may be reduced (84).

### Effect of Processing in Its Different Applications

Nutritive and bioactive components of insects can change due to pH, oxygen, light, and temperature or combinations of these during food processing. The effect of diverse cooking methods (panfrying, boiling, oven cooking, and vacuum cooking) on the nutrients of mealworms (*T. molitor* L.) has been investigated as the heating process could have deleterious effects on protein and lipid quality (15). Boiling and cooking under a vacuum maintained high protein levels (43%) and fatty acids (polyunsaturated) (30%) of raw mealworms (Table 3). Except for pan-fried mealworms, which had the highest lipid content (65%) and the lowest protein content (27%), cooking method-related changes had very little effect on macronutrient content. Cooking slightly altered the fatty acid composition of mealworms, primarily by decreasing their level of saturated fatty acids, but it also increased mealworms *in vitro* crude protein digestibility. Proteins extracted from raw and fried mealworms were less digestible than proteins extracted from all other treatments.

**Table 3:** Effect of processing insects on their different applications.

**Insect**	**Treatment**	**Effect of processing**	**References**
Cricket flour *(Acheta domesticus)*	Baked at 180 °C for 30 min.	Improved antioxidant capacity (8%) and increased protein bioaccesibility *in vitro*	(40)
African Palm Weevil *(Rhynchophorus phoenicis)* larvae	Frozen (0–90 days, −18°C) and refrigerated (0–7 days, 4°C).	The lipid oxidation of larvae was increased by sun drying, boiling, and roasting.	(85)
	Smoked (6 h), sun (5 days) and oven dried (50°C, 48 h).	The acidity of the oil was increased by boiling, refrigeration (3 days), freezing, sun drying, and electrical drying.	
	Boiled (20 min), grilled (10 min, 135°C), and deep roasted (10 min, 95°C).	The best methods for lipid preservation are refrigeration (3 days) or freezing (30 days) and smoking.	
Mealworms (*Tenebrio molitor* L.)	Grinding, defatting and isoelectric precipitation of proteins.	Its high fat content limited grinding frozen or dried larvae into flour. Defatting and isoelectric precipitation increased the concentration of *T. molitor* proteins by 11 and 15%, respectively	(86)
Mealworms (*T. molitor* L.)	Vacuum- cooked (74.0°C, 60 min), Pan-fried (1 min in 15.0 ml of olive oil), boiled (100°C, 1 min), and 15- and 30-min oven-cooked (70°C).	Decreased saturated fatty acids. *In vitro* crude protein digestibility was increased. Boiling and vacuum cooking preserved raw mealworms' high levels of protein (43%) and polyunsaturated fatty acids (30%). Pan-fried mealworms, exhibited the highest lipid content (65%) and lowest protein content (27%)	(15)
Mealworms (*T. molitor* L.)	Baked at 150°C for 10 min and boiled at 100°C for 10 min.	In boiled and baked mealworms, there were 33 and 13% more peptides, respectively than in its raw counterpart but Lipoxygenase (LOX) Inhibitory Activity and Cyclooxygenase (COX) inhibitory activity was compromised. Baked proteins exhibited higher Fe^2+^ chelation ability and radical (DPPH) scavenge activit	(16)
Locust *(Schistocerca gregaria)*		The peptides derived from boiled locust demonstrated the greatest Fe^2+^ chelation ability (IC_50_:2.57 μg/mL) and lipoxygenase and cyclooxygenase-2 inhibitory activity (IC_50_:3.13 μg/mL and 5.05 μg/mL, respectively).	
Cricket *(Gryllodes sigillatus)*		Baked cricket hydrolysates had the highest antiradical activity against DPPH (IC_50_:value 10.9 μg/mL).	
Cricket *(Gryllodes sigillatus)*	Alcalase hydrolysis [50 °C, 0.5–3% (w/w) and 30–90 min].	When compared to unhydrolyzed cricket protein, the protein solubility of hydrolysates improved, with >30% soluble protein at pH 3 and 7 and 50–90% at alkaline pH.	(35)
Locust *(Locusta migratoria)*	Enzyme hydrolysis (50°C and pH 8.0).	The protein solubility of enzyme hydrolysates was increased over a wide pH range, from 10–22 to 100% at alkaline conditions (pH 9). When compared to raw *Locusta migratoria* proteins, hydrolysis resulted in increased emulsifying activity (54%) at pH 7, improved foamability (326%) at pH 3, and improved oil binding capacity.	(87)

Likewise, lipid stability of African-consumed *R. phoenicis* larvae was evaluated at different periods of freezing (0–90 days) and refrigeration (0–7 days) storage conditions, dehydration treatments (smoke, sun, and oven dried), and cooking methods (boiled, grilled, and deep roasted) (Table 3) (85). The lipid oxidation of larvae was increased by boiling, sun drying, and roasting. The acidity of the oil was increased by boiling, refrigeration for 3 or more days, freezing, sun drying, and electrical drying. Culinary and dehydration methods significantly increased the peroxide value, especially when dehydration was followed by boiling. Cold storage (refrigeration for <3 days or freezing for less than a month) and smoking were discovered to be the best methods for lipid preservation.

The effect of heat processing on the biological activities (antioxidant and anti-inflammatory) of bioactive peptides of edible locusts (*S. gregaria)*, mealworms (*T. molitor)*, and crickets (*G. sigillatus)* was also evaluated (52). Each species of insects was boiled or baked, their peptides were extracted, and their biological activities were tested (Table 3). In boiled and baked mealworms, there were 33 and 13% more peptides, respectively, than in their raw counterparts. The effect of temperature on the bioactivities of the peptides depends on a combination of the type of process, type of an insect, and a bioactivity assay. A peptide fraction from baked cricket *G. sigillatus* hydrolysate demonstrated the highest antiradical activity against DPPH (IC_50_: 10.9 μg/ml). The peptides derived from boiled locust *S. gregaria* hydrolysate demonstrated the greatest Fe^2+^chelation ability (IC_50_: 2.57 μg/ml). The most potent lipoxygenase and cyclooxygenase-2 inhibitory activity (IC_50_: 3.13 and 5.05 μg/ml, respectively) were found in the peptide fraction of a protein preparation from the locust *S. gregaria*. Moreover, thermal processing of cricket (*A. domesticus*) flour (cooked and baked) had a significantly (*p* < 0.05) improved antioxidant activity (Table 3); this could be due to conformational changes in cricket proteins, exposing proton-donating residues, specifically cysteine (40). Baking increased the gastric proteolysis and bioaccessibility of cricket proteins (40).

Not only the insects, either whole or in flour, can be consumed but they also can be processed to generate other ingredients, such as protein isolates suitable for the development of new food formulations, and potentially substitute conventional proteins used in food/feed formulations. Processes to generate protein isolates of insects may include centrifugal fractionation, pH protein solubilization, salt solubilization, enzyme hydrolysis, and temperature (35, 86–88). The effect of extraction procedures for recovering insect protein on techno-functional and compositional properties has been investigated. *T. molitor* protein content ranges around 39% and concentrates obtained from this insect had a final protein content of 60% (89). The processability of *T. molitor* larvae was limited (Table 3) by its high-fat content (86). *T. molitor* protein concentration increased by 11 and 15%, respectively, after defatting and isoelectric precipitation (86).

*L. migratoria* protein content ranges around 66%, and concentrates obtained from this insect had a final protein content of 82%, showing maximum solubility at pH 9 (100%) (87). The protein solubility of enzyme hydrolysates was increased over a wide pH range, from 10–22% to 100% at alkaline conditions (pH 9) (87, 88). In terms of emulsifying activity at pH 5, foamability at pH 3 and 3% sodium chloride (NaCl), and foam stability at pH 9, *L. migratoria* proteins performed similarly to egg white protein (87). When compared with raw *L. migratoria* proteins, hydrolysis resulted in increased emulsifying activity at pH 7 (54%), improved foamability at pH 3 (326%), and advanced oil-binding capacity (88).

Whole crickets (*G. sigillatus*) were hydrolyzed with alcalase and outperformed unhydrolyzed cricket protein in emulsifying and foaming properties (Table 3) (35). When compared with the control, protein solubility of hydrolysates improved, with >30% soluble protein at pH 3 and 7 and 50–90% at alkaline pH; hydrolyzed protein of crickets can be introduced in acidic food systems (e.g., sports drinks) (35). Foamability ranged from 100 to 155% with high foaming capacity and foam stability (99%, 92%) (35, 90). *G. sigillatus* protein concentrate had noticeable water and oil-holding capacity (3.33 g/g) (90). Future research should focus on developing processes to isolate and recover a proteic, a lipidic and a fibrous fraction to be used in food formulations.

## Adoption of Insect-Based Food Barriers

### Consumer Perception of Entomophagy

The consumption of insect-based foods is a complex situation powered by several factors mainly because insects are not, for many people, traditional ingredients of the diet. In one part, insect-based products are commercialized as gourmet products directed to a specific market of persons that are ready to or may be interested in consuming these exotic products. These persons are looking for different flavors in their meals with the additional advantage but not the main one of having an enhanced nutrimental product. On the other side, there are some countries where insects are already directly consumed by persons as another choice of a protein source, mainly due to the limited access to high-protein content food. Then, some insect-based products are developed in order to improve protein composition on conventional highly consumed products and are directed to persons that could present some nutritional deficiency. On another side, there is a growing interest in the consumption of insect-based foods as a result of the growing demand for alternative protein sources. Considering the high content of protein of insects and their low environmental impact, it results in a very attractive option directed mainly to western societies of developed countries where insects are not actual options in their consumption in the human diet. Therefore, a great quantity of studies and products is developed, looking forward to the increasing consumption of these insect-based foods with the arguments of health benefit and low environmental impact.

One of the most difficult aspects of introducing insects into the human diet is changing consumer perception. Although there is a growing interest in commercializing insect-based foods due to different reasons stated before, it exits known unwillingness from the society to broadly accept insect-based foods. In contrast, there are some regions, mostly from Africa, East Asia, Latin America, and Australia, where insects are a part of the basic and traditional diet (91). For example, Africa counts with the highest number of countries where insects are consumed (36 countries), followed by Asia (29 countries), America (23 countries), and Europe (11 countries) (92). In contrast, in Western countries, specifically Western Europeans, Canadians, non-Aboriginal Americans, New Zealanders, and Western Australians, insects are not currently widely consumed.

**Figure 1:**
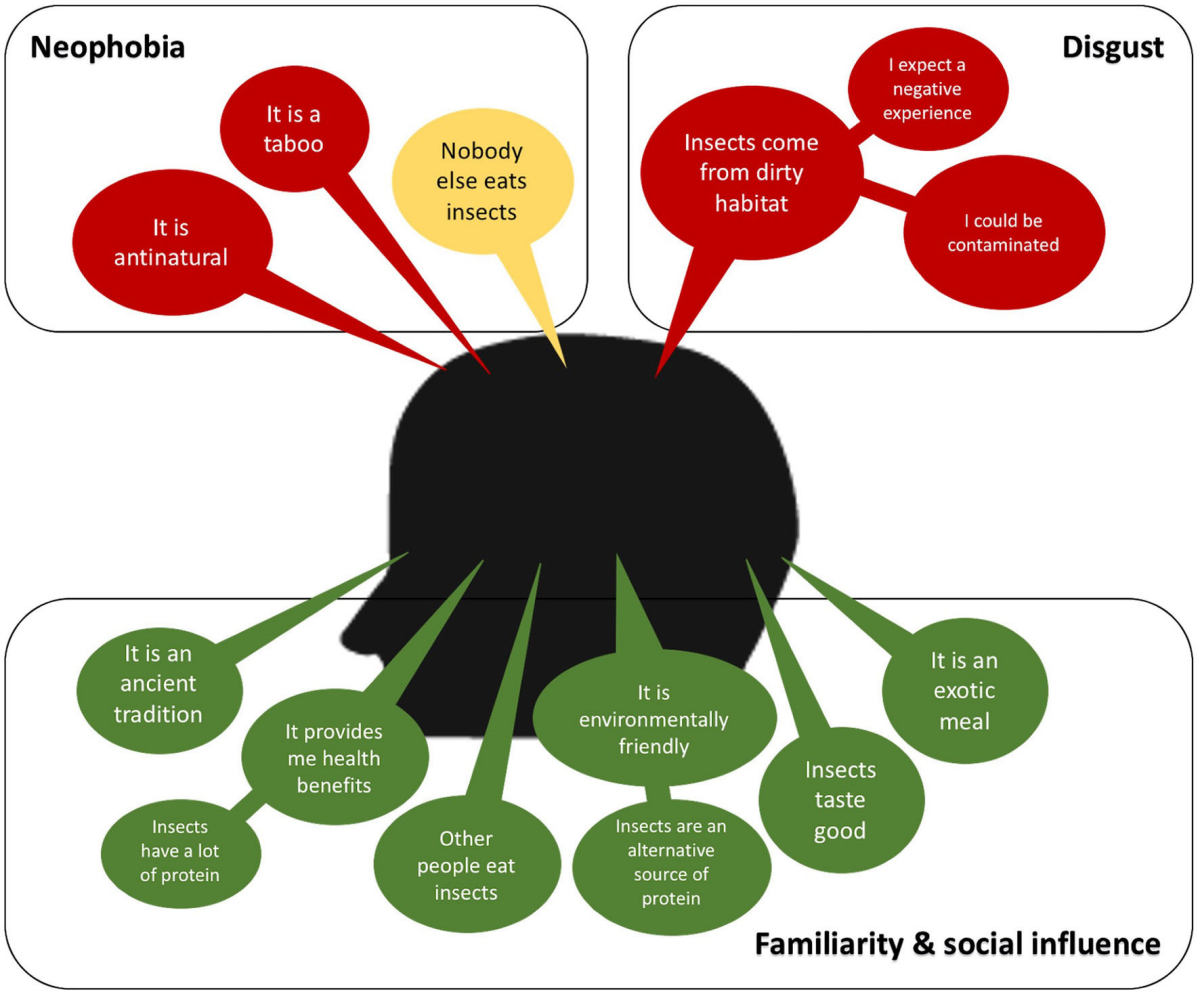
A consumer-perception diagram. A positive consumer-perception is colored in green, neutral is in yellow and negative is in red.

Eating insects can be considered a taboo, an ancient tradition exclusive of indigenous people (tropical rural areas), and antinatural according to social norms and beliefs of Western countries (93, 94). In Figure 1, some of the positive and negative main ideas boarding the perception of the consumers are presented. The most frequent factors found in the literature that explain this unwillingness to insect-based food are food neophobia and disgust (94–97). Food neophobia is the fear of eating unfamiliar foods, and it is directly dependent on the culture and social norms. Then the rejection to eat insects can be diminished by increasing the familiarity of insect-based foods, which can be achieved with strategies that are discussed later. On the other hand, the disgust at eating insects has been reported as a reaction resulted from an expected negative consumption experience, an expected feeling of discomfort due to nature, mostly dirty, of the habitats of insects, a fear of contamination and disease (94, 95, 98, 99). Therefore, depending on the main factors of the unwillingness reaction to eat insect-based foods should be the strategy to encourage its consumption.

Studies from different perspectives such as psychological, gastronomical, marketing, and among others on different segments of Western societies have been reported (100–103). These studies were directed to find the reasons for this unwillingness to eat insects and propose strategies to encourage Western people to include insects in their diet. A study with a psychological perspective explains that people tend to select their food driven by taste preferences rather than rational decisions. Then they propose an alternative sensorial-driven strategy to make people eat insects regularly. In this way, people will have a psychologically realistic motivation and gastronomic interest (100). Another study (101) related to entomophagy on attitudes of Western Australian seniors found disgusting reactions and concerns about the safety of the food as negative attitudes toward eating insects. These are implicit associations that were also discussed previously (95). An important reason related to the disgusting reaction is that Western people considered entomophagy as incompatible with their cultural beliefs and values. Therefore, a strategy (101) proposed is to guarantee food safety and disguising insects as food. Other authors (103) studied the effect of using mindfulness on the willingness to eat insects on young people of Americans and Australians. They discovered that mindfulness increases disgust and decreases willingness to try eating insects as a result of the increased state of awareness that mindfulness induces.

Other studies relate the social influence on eating insects (98) and present the results of the effects of concerns about food contamination and a perceived social eating norm. It reveals that there is no link between perceived infectability and insect-eating disgust, but social norms have a significant impact on the willingness of people to eat insects. As a result, food culture and perceived social norms play important roles in explaining why people eat strange and possibly disgusting foods (98). There is another study where the perception of people that consume an insect-based diet is considered. They discovered that people perceive insect consumers to be more health-conscious, environmentally friendly, imaginative, brave, interesting, and knowledgeable than meat consumers. The social influence of people who consume such products may then be significant (99).

Also, there are some reported efforts to improve the gastronomic experience of eating insects. For example, evaluation of the sensory and physiological properties of a broth made from frozen crickets and alive crickets when cooked to obtain a processing method with best results according to the sensory evaluation of the food (102). In the same way, insects that are soft and juicy after being boiled or vacuum cooked are less preferred than crispy insects produced by oven cooking or frying (15).

Sensory attributes of insect-based foods depend on their diet. Insects take the flavor of the additives and ingredients they consume. For example, the exoskeleton of insects has an impact on texture, specifically on crunchiness. This characteristic helps consumers appreciate the crackers-like texture of food prepared with insects with crunchy exoskeletons. In their younger stages, chitins are lower than in older insects; this makes the products less crispy, but it can help to improve their digestibility. Another characteristic is the color, which can change with the cooking process; it can be gray, brown, red, or black. Because of this change, it is necessary to carefully choose the type of an insect and the processing applied in the final product (104).

Although, in the case of introducing insects to the diet, it is suggested to use processed insects in order to make them no visible in the food. There are many studies that proposed very similar strategies to increase the acceptance and regular consumption of insect-based foods. It is well-known that a sole strategy would fail, such as what happens nowadays (105). Therefore, an integrative strategy must be considered. First, it is imperative to generate positive associations to overcome negative emotions toward insect-based foods, using, for example, marketing and social influence. Along with this, it is important to introduce contextual cultural information about insects, for example, the benefits of insect consumption on human health and the environment. In this way, it is expected to harness social norms and increase familiarity with the consumption of insect-based foods (93, 96–99, 105, 106). Although marketing is not enough, an appropriate product design must be done, considering a product made from processed insects or whole insects, with good sensory qualities, guaranteeing safety and compatibility with local food (93, 99, 106). It would be expected that the acceptance of insect-based foods increases the acceptance of unprocessed insects in Western countries as well.

### Insect Food Safety and Regulation

#### Microbial Hazards

For many years, experiences of entomophagy have been widespread in traditional Africa, Asia, Oceania, and South America cultures; in fact, about two billion people already consume edible insects every day. However, the sanitary conditions of these insects are not always adequate due to a lack of information, control, and regulation of the risks derived from their consumption. Insect biodiversity is huge, implying a remarkable diversity of metabolic pathways and microbiomes among the various species (107), making the process of hazard identification complicated. Other factors, such as cultivation, processing, and further storage conditions, in addition to the own characteristics of insects, heavily influence the food safety of edible insects and are factors that affect the risk assessment of insect consumption as feed and food. To ensure the safety of insects and their products, processing and storage must adhere to the same health and sanitation regulations as any other food.

Microbial hazards are one of the most serious food safety issues. In the last years, several studies have been conducted to investigate the microbial composition of various edible insects by two different approaches: through classical food microbiology methods (microbial counts) as well as through the next generation sequencing methods (NGS). The first approach has the aim of quantifying the viable cells in order to develop microbial criteria for edible insects similar to those used for other food products and, later, as a way to investigate qualitatively microbial community composition, although viable non-culturable or non-viable bacteria will be detected.

Table 4 shows a review of the most significant results on microbial composition organized by the most accepted insect species in food and feed. Independently, of the insects studied, general microbial contamination, analyzed as a total mesophilic aerobes, is very high (7–8 Log_10_ CFU/g), similar to that usually found in raw vegetables and fresh herbs rather than counts found in any other animal product (110). At the same time, fresh insects also have a high enterobacteriaceae microbiological load (4.2–8 Log_10_ CFU/g), bacterial spores (1.3–4.8 Log_10_ CFU/g), and presumptive yeast and molds (4.2–7.2 Log_10_ CFU/g). Because enterobacteriaceae are hygiene indicators, the presence of this microbial group in a given food or feed should be carefully evaluated. Several explanations could be found for those values. From one side is the fact that the vast majority of insects are consumed whole, including their guts, which could contain 10^6^–10^12^ CFU/g (107). To decrease these values, some producers subject the insects to a fasting period of 24–48 h prior to slaughter. However, the effectiveness of this technique to reach microbial concentration below risk levels is not yet proven. Other factors that could have an impact on the presence and growth of potential spoilage bacteria and food pathogens are rearing conditions and poor processing in combination with long shipping transportation.

**Table 4:** Microbial contamination [Log_10_ (N) (CFU/g)] reported for different insects.

**Insect**	**Microorganism concentration [Log**_****10****_**(N) CFU/g]**								**References**
	**Total mesophillic aerobes**	**Total aerobic spores**	**Enterobacteriaceae**	**Lactic acid bacteria (LAB)**	***Salmonella* spp**.	***Listeria monocytogenes***	**Yeast and molds**	***Bacillus cereus***	
Black soldier fly *(Hermetia illucens)*	7.19	–	6.06	–	–	ND	–	–	(108)
	5.28	–	4.29	–	4.04	ND	5.19	–	(109)
Mealworm *(Tenebrio molitor)*	2.5–5.6	2.2–7	–	–	–	–	–	2–4.7	(110)
	8.5–8.2	3–3.7	7.1–6.1	8.2–7.7	ND	ND	–	–	(111)
	7.7–8.3	1–3.5	6.8–7.6	7–7.6	–	–	5.2–5.7		(112)
	8–9.3	1.7–4.2	6.9–7.8	7.3–8.2	ND	ND	4.2–6	ND	(113)
	7.7	2.1	6.8	–	–	–	–	–	(114)
House cricket *(Acheta domesticus)*	1.6–8	2–8	–	–	–	–	–	2–4.7	(110)
	8.1–8.7	2.6–4.1	7.2–8.0	7.4–8.3	–	–	5.6–7.2		(113)
	4.32	–	ND	–	–	–	4.89	–	(115)
				–	–	–		–	(111)
	7.49–7.69	3.5	6.69–7	–	ND	ND	5.39	–	(116)
	7.97	–	–	–	–	–	4.80	–	(117)
	7.2	3.6	4.20	–	–	–	–	–	(114)
Grasshopper *(Ruspolia differens)*	8.38–9.41	3.75–4.87	6.89–7.83	7.99–9.11	–	–	5.77–7.12	–	(118)
Grasshopper *(Locusta migratoria migratorioides)*	7.8–8.6	3.3–3.8	7.6–7.1	7.6–8.5	–	–	5–5.4	–	(112)

*ND, not detected*.

As far as microbial pathogens are concerned, no *L. monocytogenes* has been detected in any of the samples described in the literature; however, the presence of *Salmonella* spp. and *Bacullis cereus* in some samples are registered (108, 110). The development and the type of safety regulation applied related to insect consumption are very variable for different regions. Some countries with traditional insect consumption such as Thailand have developed guidance on good agricultural farming practices (119), and other countries such as Belgium or The Netherlands advise producers to refer to the EU Regulation's hygiene criteria for minced meat (EC 1441/2007). On average, the level of microbial contamination of fresh insects is above the limits established by this regulation, and some pathogens such as *Salmonella* spp. or *B. cereus* have been described in the literature; therefore, to avoid or reduce the risks associated with the consumption of edible insects, a processing step with an antimicrobial effect is required.

While classical methods based on microbial counts are necessary to establish food hygiene criteria, efficient and in-depth methods to evaluate the biodiversity of insect microbiome are also of utmost importance. Several next-generation high throughput sequencing (NGS) techniques have been used to study the microbiota of insects in the absence of culture. Great bacterial diversity and variation between different insects, even among the same insects reared in different facilities, were seen. When studying *A. domesticus* microbiota, it has been suggested that microbial communities are highly dependent on rearing conditions and are heavily influenced by dietary and environmental factors such as breeder manipulation, food and water microbiota, and so on (107). From the food safety perspective, some authors pointed out the high occurrence of *Enterobacteriaceae*, with the closest relatives of the genus *Enterobacter* and lactic acid bacteria with the closest relatives to *Enterococcus* and *Lactococcus* species, dominating in all samples (111, 115). Spore-forming bacteria from the genera *Anaerobacillus, Bacillus, Clostridium, Paenibacillus*, and *Geobacillus* are also remarkable insect microbiota components as described before (111–113). Other spoilage and pathogenic microorganisms assigned to genera *Weissella, Pediococcus, Streptomyces, Acinetobacter, Agrococcus, Naxibacter, Arthrobacter, Planomicrobium, Rufibacter, Bacillus, Clostridium, Vibrio, Desulfovibrio, Aspergillus, Loktanella, Escherichia, Tetrapisispora, Eurotium, Debaryomyces*, and *Wallemia* were identified by PCR-DGGE in *A. domesticus, L. migratoria*, and *T. molitor* (120). In general, a wide range of potential spoilage bacteria and food pathogens may be present in insects, making them vectors for foodborne diseases; therefore, to avoid or reduce the risks associated with insect consumption, a microbiocidal processing step is required. Like other foods, these risks can be mitigated by employing proper hygiene and manufacturing practices.

#### Preservation Processes to Guarantee Microbial Safety

To guarantee food safety while increasing the shelf life of edible insects, food preservation techniques should be applied to control growth and/or inactivate spoilage and pathogen microorganisms. Decreasing water content (drying, freeze-drying), acidification or thermal treatment (boiling, blanching, or sterilizing) of foods is the most common food preservation strategy applied.

Regarding the thermal treatments, different combinations of cooking, boiling, and drying to inactivate the flora of *T. molitor* and different crickets (*A. domesticus* and *Gryllus bimaculatus*) were tested (114, 121). The treatment of 30-min cooking + 12-h drying at 80 and 100°C and 5-min boiling + 24-h drying at 55°C was the most efficient to inactivate the microbial flora (enterobacteria, yeast and molds, staphylococci); however, these treatments were not enough to inactivate spore-forming bacteria. A tyndalization-like treatment reduced all microbial counts below detection limits, but food quality (mainly physical-chemical properties and nutrients) should be considered. Blanching (99°C for 5–10 min) and sterilization (120°C, 16 min) were also considered as sanitation methods to reduce microbial contamination in *T. molitor* and *A. domesticus* (117). Both treatments were enough to diminish enterobacteriaceae and yeast and molds below the limits for fresh minced meat (Belgian requirements), but only sterilization succeeds for spore-forming bacteria. The same authors pointed out freeze-drying as a useful preservation technique. However, because this technique is known to mostly inactivate microorganisms rather than kill them, it is necessary to heat-treat freeze-dry insects before eating them (121). Different household cooking techniques (oven-cooking, panfrying, vacuum-cooking, and boiling) were investigated to inactivate total aerobic flora (15). Only vacuum-cooking, boiling, and frying reduced the microbial flora to less than the lower limit for fresh minced meat, with boiling being the most effective treatment. Other authors (108, 109) have considered high hydrostatic pressure treatment (HPP) as an alternative preservation method to reduce a microbial load in *H. illucens*. The treatment of 400 MPa during 7 min was effective in inactivation of pathogen *Escherichia coli* O157:H7 and *S. typhimurium* in more than 5Log_10_, while a low reduction was reached in total mesophilic flora, probably due to the presence of spore-forming bacteria and the incapacity of HPP to inactive them.

From the studies presented in the literature, it can be deduced that insects, like many foods, can be a vector for foodborne disease transmission. Nevertheless, these risks can be reduced by optimizing and controlling the production and processing conditions. Furthermore, regulations and policies governing the safety of edible insects will be a valuable tool in improving the safety of insects as food and feed (122).

#### Allergen Risk

Some studies have reported cases of anaphylactic reactions, following the consumption of edible insects; these reactions, perhaps, were caused by the existing relationship between insects and arthropods (including shrimps, dust mites, etc.) (123). Purines, which are found in proteins, are made up of hydrogen, carbon, and nitrogen, as well as adenine and guanine. Uric acid is a byproduct of purine metabolism in the human body, which can be harmful to people with gout. Insects, such as mealworms, house crickets, discoid roaches, and desert locusts, were studied to see if there are sex differences in purine derivate content. Significative differences between sexes were found in house crickets and desert locusts; for house crickets, male samples showed guanine and hypoxanthine values of 1.66 and 2.04 g/kg dry matter, respectively, while, for females, the values were 1.37- and 1.85-g/kg dry matter. For male desert locusts, the values were 1.15- and 0.65-g (kg dry matter) guanine and hypoxanthine, respectively, while female desert locusts showed values of 1.15- and 0.65-g/kg dry matter. The highest adenine values were found in desert locusts—for males, 1.95-g/kg dry matter, while for females, 2.73-g/kg dry matter (124). Humans can consume insect species with no additional risks when compared with commonly consumed animal products.

#### Regulation

Insects might be a source of biological and chemical contaminants that could affect the health of consumers (125). In fact, that has been the focus of Safety Agencies like the European Food Safety Authority (EFSA), which concluded that the health risks associated with insects consumption depend on the species and substrate used for farming, the way of reared, harvested, and processed (126, 127). Several European countries, including Belgium, Switzerland, and the Netherlands, have declared that certain insects are permissible for production and consumption (91). In 2014, in Belgium, the Scientific Committee of the Federal Agency for the Safety of the Food Chain agreed that farmed insects can be consumed or put on to market previously processed with a heating step (128). Likewise, the Swiss Federal Food Safety and Veterinary Office stated that they support the sale of crickets, grasshoppers, and mealworms (91). In Europe, since November 2015, insects have been designated as novel foods and are subject to a centralized procedure governed by EU Regulation 2015/2283. For instance, processed animal proteins (blood products, gelatin, collagen, and hydrolyzed proteins derived from ruminants) cannot be used as a substrate for insects to prevent pathologies related to spongiform encephalopathies (129). Exception to that are seven insect species (*H. illucens, Musca domestica, T. molitor, A. diaperinus, A. domesticus, G. sigillatus*, and *Gryllus assimilis*) that could be used in aquaculture and pet food in the European Union.

Despite consuming a high number of insect species in Africa, there is lack of specific regulations in many of them (130). Exceptions are the Botswana's law that considered mopane caterpillars (*Imbrasia belina*) as edible (130), and, more recently, the Kenya Bureau of Standards (KEBS) has approved three national standards for guiding the primary production of edible insects and also how to handle the resulting by-products. Specifically, standards describe the requirements for infrastructure and environmental issues (131) or for the food and feed products containing edible insects (131, 132).

In the United States, insects can be sold as food if they are raised by companies that have set up special production lines for human consumption and processed under sanitary conditions, following current good manufacturing practices and must be properly labeled according to FDA regulations (91). In Oceania, the Food Standards Australia New Zealand (FSANZ) Advisory Committee on Novel Foods (ACNF), does not identify any safety concerns for *Z. morio, T. molitor*, and *A. domestica* that are considered non-traditional food but only in the case of individuals with crustaceans allergies. In Asia, there is a long tradition in consuming insects, and, generally, they do not have specific regulations, although, for instance, in China, there are local standards for some insects. For instance, there are safety standards for edible frozen fresh silkworm pupae in Guangxi Zhuang Autonomous Region (133). Then legislation around insect-based foods is being developed as the interest in their consumption is growing. The food safety of edible insects can thus contribute to the process of accepting insects as an alternative food source, changing perceptions of entomophagy of developed countries.

## Conclusions

When insects are used as a whole food or as ingredients, they have positive impacts on nutritional value by increasing the quantity and quality of proteins, fats, and micronutrients. The enrichment of staple cereal-based food with insects increases the proportions of protein and fat and is feasible and convenient to prevent nutritional deficiencies in populations that have difficult access to protein-rich food. By using insects as ingredients, the organoleptic qualities (taste, appearance, texture, color, etc.) nor the technological properties of the products are compromised. Similarly, bioactive compounds found in insects have been shown to benefit a variety of diseases, such as coronary heart disease, cancer, and inflammation. Bioactive-derived insect compounds should be used to design foods for improved health. The adoption of an insect-based food primary barrier is consumer perception of entomophagy. Although, in the case of introducing insects to the diet, it is suggested to use processed insects in order to make them no visible in the food. Likewise, in comparison to commonly consumed animal products, insect species can be consumed by humans with no additional risks. The food safety of edible insects can thus contribute to the process of accepting insects as an alternative food source, changing the perceptions of developed countries of entomophagy. Although insects are consumed by 30% of the population of the world, there is still a long way to go to be fully accepted, having regulations will allow insects-based foods to be so common in the future that we cannot understand why we did not incorporate them into our diet before these slimy yet satisfying ingredients.

## Author Contributions

CI-H and BA-E contributed to conception and design of the study. BA-E and AR organized the database. BA-E wrote the first draft of the manuscript. CI-H, AR, CR, and DR wrote sections of the manuscript. All authors contributed to manuscript revision, read, and approved the submitted version.

## Conflict of Interest

The authors declare that the research was conducted in the absence of any commercial or financial relationships that could be construed as a potential conflict of interest.

## References

[1] FAO. Land Use in Agriculture by the Numbers. Rome: Food and Agriculture Organization of the United Nations (2020).

[2] Beusen AHW Bouwman AF Heuberger PSC Van Drecht G Van Der Hoek KW. Bottom-up uncertainty estimates of global ammonia emissions from global agricultural production systems. Atmosph Environ. (2008) 42:6067–77. 10.1016/j.atmosenv.2008.03.044

[3] van Huis A Van Itterbeeck J Klunder H Mertens E Halloran A Muir G. Edible Insects: Future Prospects for Food and Feed Security (No. 171). Rome: Food and Agriculture Organization of the United Nations (2013). p. 201.

[4] de Castro RJS Ohara A Aguilar JGdS Domingues MAF. Nutritional, functional and biological properties of insect proteins: processes for obtaining, consumption and future challenges. Trends Food Sci Technol. (2018) 76:82–9. 10.1016/j.tifs.2018.04.006

[5] Belluco S Losasso C Maggioletti M Alonzi C Paoletti M Ricci A. Edible insects in a food safety and nutritional perspective: a Critical review. Compreh Rev Food Sci Food Safety. (2013) 12:296–313. 10.1111/1541-4337.12014

[6] Rumpold BA Schlüter OK. Potential and challenges of insects as an innovative source for food and feed production. Innovat Food Sci Emerg Technol. (2013) 17:1–11. 10.1016/j.ifset.2012.11.005

[7] Oonincx DGAB Van Itterbeeck J Heetkamp MJW Van Den Brand H Van Loon JJA Van Huis A. An exploration on greenhouse gas and ammonia production by insect species suitable for animal or human consumption. PLoS ONE. (2010) 5:e14445. 10.1371/journal.pone.0014445 21206900 PMC3012052

[8] Jonas Levi A Martinez J-JI. The high level of protein content reported in insects for food and feed is overestimated. J Food Comp Analysis. (2017) 62:4. 10.1016/j.jfca.2017.06.004

[9] Jolly A Agea J Obaa B James O Dorothy N. Process development, sensory and nutritional evaluation of honey spread enriched with edible insects flour. Afr J Food Sci. (2017) 11:30–9. 10.5897/AJFS2016.1463

[10] de Oliveira LM da Silva Lucas AJ Cadaval CL Mellado MS. Bread enriched with flour from cinereous cockroach (*Nauphoeta cinerea*). Innovat Food Sci Emerg Technol. (2017) 44:30–5. 10.1016/j.ifset.2017.08.015

[11] Kim HW Setyabrata D Lee Y Jones O Kim Y. Effect of house cricket (*Acheta domesticus*) flour addition on physicochemical and textural properties of meat emulsion under various formulations. J Food Sci. (2017) 12:2787–93. 10.1111/1750-3841.13960 29095501

[12] Ottoboni M Spranghers T Pinotti L Baldi A Jaeghere W Eeckhout M. Inclusion of *Hermetia illucens* larvae or prepupae in an experimental extruded feed: process optimisation and impact on *in vitro* digestibility. Ital J Anim Sci. (2017) 17:1–10. 10.1080/1828051X.2017.1372698

[13] Cheseto X Kuate SP Tchouassi D Ndungu M Teal P Torto B. Potential of the desert *locust schistocerca* gregaria (Orthoptera: acrididae) as an unconventional source of dietary and therapeutic sterols. PLoS ONE. (2015) 10:e0127171. 10.1371/journal.pone.0127171 25970517 PMC4429980

[14] Kaur KD Jha A Sabikhi L Singh AK. Significance of coarse cereals in health and nutrition: a review. J Food Sci Technol. (2014) 51:1429–41. 10.1007/s13197-011-0612-9 25114333 PMC4108649

[15] Caparros Megido R Poelaert C Ernens M Liotta M Blecker C Danthine S. Effect of household cooking techniques on the microbiological load and the nutritional quality of mealworms (*Tenebrio molitor* l. 1758). Food Res Int. (2018) 106:503–8. 10.1016/j.foodres.2018.01.002 29579954

[16] Zielińska E Baraniak B Karaś M. Antioxidant and anti-Inflammatory activities of hydrolysates and peptide fractions obtained by enzymatic hydrolysis of selected heat-Treated edible insects. Nutrients. (2017) 9:970. 10.3390/nu9090970 28869499 PMC5622730

[17] Azzollini D Derossi A Fogliano V Lakemond CMM Severini C. Effects of formulation and process conditions on microstructure, texture and digestibility of extruded insect-riched snacks. Innovat Food Sci Emerg Technol. (2018) 45:344–53. 10.1016/j.ifset.2017.11.017

[18] Ranum P Pena-Rosas J Garcia-Casal MN. Global Maize Production, Utilization, and Consumption. Annals of the New York Academy of Sciences. (2014). 1312:105–12. 10.1111/nyas.12396 24650320

[19] Serna-Saldívar SO. Cereal Grains. Properties, Processing, and Nutritional Attributes. 1st ed. Boca Raton FL; CRC Press (2010).

[20] González CM Garzón R Rosell CM. Insects as ingredients for bakery goods. A comparison study of *H. illucens, A. domestica* and *T. molitor* flours. Innovat Food Sci Emerg Technol. (2019) 51:205–10. 10.1016/j.ifset.2018.03.021

[21] Aguilar-Miranda ED López MG Escamilla-Santana C Barba de la Rosa AP. Characteristics of maize flour tortilla supplemented with ground *Tenebrio molitor* larvae. J Agric Food Chem. (2002) 50:192–5. 10.1021/jf010691y 11754566

[22] Cuj-Laines R Hernández-Santos B Reyes-Jaquez D Delgado-Licon E Juárez-Barrientos JM Rodríguez-Miranda J. Physicochemical properties of ready-to-eat extruded nixtamalized maize-based snacks enriched with grasshopper. Int J Food Sci Techn. (2018) 53:1889–95. 10.1111/ijfs.13774

[23] Adeboye AO Bolaji TA Fatola OL. Nutritional composition and sensory evaluation of cookies made from wheat and palm weevil larvae flour blends. Ann Food Sci Techn. (2016) 17:543–7. Available online at: www.afst.valahia.ro

[24] Kim H-W Setyabrata D Lee YJ Jones OG Kim YHB. Pre-treated mealworm larvae and silkworm pupae as a novel protein ingredient in emulsion sausages. Innovat Food Sci Emerg Technol. (2016) 38:116–23. 10.1016/j.ifset.2016.09.023

[25] Smetana S Larki NA Pernutz C Franke K Bindrich U Toepfl S. Structure design of insect-based meat analogs with high-moisture extrusion. J Food Eng. (2018) 229:83–5. 10.1016/j.jfoodeng.2017.06.035

[26] Cho J-H Zhao H-L Kim J-S Kim S-H Chung C-H. Characteristics of fermented seasoning sauces using *Tenebrio molitor larvae*. Innovat Food Sci Emerg Technol. (2018) 45:186–95. 10.1016/j.ifset.2017.10.010

[27] Zhao X Cheng Q Qian Y Yi R Gu L Wang S. Insect tea attenuates hydrochloric acid and ethanol-induced mice acute gastric injury. Exp Therap Med. (2017) 14:5135–42. 10.3892/etm.2017.5181 29201228 PMC5704295

[28] Irungu FG Mutungi CM Faraj AK Affognon H Tanga C Ekesi S. Minerals content of extruded fish feeds containing cricket (*Acheta domesticus*) and black soldier fly larvae (*Hermetia illucens*) fractions. Int Aquatic Res. (2018) 10:101–3. 10.1007/s40071-018-0191-8

[29] Reuters in Helsinki. Anyone for crickets? Finnish bakery sells bread made from insect. Finland. (2017). Available online at: https://www.theguardian.com/world/2017/nov/23/anyone-for-crickets-brSernaead-made-insect-finnish-bakery-fazer (accessed March 21, 2021).

[30] Gmuer A Nuessli Guth J Hartmann C Siegrist M. Effects of the degree of processing of insect ingredients in snacks on expected emotional experiences and willingness to eat. Food Q Preference. (2016) 54:117–27. 10.1016/j.foodqual.2016.07.003

[31] Forbes. Insect Food Company Aspire Acquires Cricket Protein Bar maker Exo 2018. Available online at: https://www.forbes.com/sites/alexknapp/2018/03/08/cricket-food-company-aspire-acquires-cricket-protein-bar-maker-exo/#6aaa228f4f19 (accessed January 23, 2020).

[32] Ojinnaka MC Emeh TC Okorie SU. Evaluation of the quality of composite maize-wheat chinchin enriched with *Rhynchophorous phoenicis*. J Food Res. (2016) 5:26–35. 10.5539/jfr.v5n4p26

[33] Idolo I. Nutritional and quality attributes of wheat buns enriched with the larvae of *Rhynchophorus phoenicis* f. Pak J Nutr. (2010) 9:1043–6. 10.3923/pjn.2010.1043.1046

[34] Kinyuru JN Kenji GM Njoroge MS. Process development, nutrition and sensory qualities of wheat buns enriched with edible termites (*Macrotermes subhylanus*) from lake victoria region, kenya. Afric J Food Agricult Nutr Dev. (2009) 9:48411. 10.4314/ajfand.v9i8.48411

[35] Hall FG Jones OG O'Haire ME Liceaga AM. Functional properties of tropical banded cricket (*Gryllodes sigillatus*) protein hydrolysates. Food Chem. (2017) 224:414–22. 10.1016/j.foodchem.2016.11.138 28159288

[36] Belluco S Halloran A Ricci A. New protein sources and food legislation: the case of edible insects and eU law. Food Secur. (2017) 9:803–14. 10.1007/s12571-017-0704-0

[37] Iaconisi V Marono S Parisi G Gasco L Genovese L Maricchiolo G. Dietary inclusion of *Tenebrio molitor* larvae meal: effects on growth performance and final quality treats of blackspot sea bream (*Pagellus bogarave*o). Aquaculture. (2017) 476:49–58. 10.1016/j.aquaculture.2017.04.007

[38] Song SG Chi SY Tan BP Liang GL Lu BQ Dong XH. Effects of fishmeal replacement by *Tenebrio molitor* meal on growth performance, antioxidant enzyme activities and disease resistance of the juvenile pearl gentian grouper (*Epinephelus lanceolatus*♂ × *epinephelus fuscoguttatus*). Aquacult Res. (2018) 49:2210–7. 10.1111/are.13677

[39] Wang Y Miao X Sun J Cai L. Oxidative stress in diabetes: Molecular basis for diet supplementation. In Preddy VR, editor. Molecular Nutrition and Diabetes. 2nd ed. Academic Press (2016). 65–72. 10.1016/B978-0-12-801585-8.00006-3

[40] David-Birman T Raften G Lesmes U. Effects of thermal treatments on the colloidal properties, antioxidant capacity and in-vitro proteolytic degradation of cricket flour. Food Hydrocoll. (2018) 79:48–54. 10.1016/j.foodhyd.2017.11.044

[41] Kaya M Akyuz B Bulut E Sargin I Tan G Erdonmez D. DNA interaction, antitumor and antimicrobial activities of three-dimensional chitosan ring produced from the body segments of a diplopod. Carbohydr Polymers. (2016) 146:80–9. 10.1016/j.carbpol.2016.03.033 27112853

[42] Li Q Ray CS Callow NV Loman AA Islam SMM Ju LK. *Aspergillus niger* production of pectinase and alpha-galactosidase for enzymatic soy processing. Enzyme Microbial Techn. (2020) 134:7. 10.1016/j.enzmictec.2019.109476 32044023

[43] Morganti P Stoller M. Chitin and lignin: natural ingredients from waste materials to make innovative and healthy products for humans and plant. Chem Eng Trans. (2017) 60:319–24. 10.3303/CET1760054

[44] Sayari N Sila A Abdelmalek BE Abdallah RB Ellouz-Chaabouni S Bougatef A. Chitin and chitosan from the norway lobster by-products: antimicrobial and proliferative activities. Int J Biol Macromol. (2016) 87:163–71 10.1016/j.ijbiomac.2016.02.057 26920243

[45] Tan G Kaya M Tevlek A Sargin I Baran T. Antitumor activity of chitosan from mayfly with comparison to commercially available low, medium and high molecular weight chitosans. In vitro Cell Dev Biol Anim. (2018) 54:366–74. 10.1007/s11626-018-0244-8 29654403

[46] Liu S Sun J Yu L Zhang C Bi J Zhu F. Antioxidant activity and phenolic compounds of *holotrichia parallela motschulsky* extracts. Food Chem. (2012) 134:1885–91. 10.1016/j.foodchem.2012.03.091 23442634

[47] Suh H-J Kim S-R Hwang J-S Kim MJ Kim I. Antioxidant activity of aqueous methanol extracts from the lucanid beetle, *Serrognathus platymelus castanicolor* motschulsky (Coleoptera: *lucanidae*). J Asia-Pacific Entomol. (2011) 14:95–8. 10.1016/j.aspen.2010.10.002

[48] Dutta P Dey T Dihingia A Manna P Kalita J. Antioxidant and glucose metabolizing potential of edible insect, *brachytrupes orientalis* via modulating nrf2/AMPK/GLUT4 signaling pathway. Biomed Pharm. (2017) 95:556–63. 10.1016/j.biopha.2017.08.094 28869893

[49] Seo M Kim J Moon S-S Hwang J-S Kim M-A. Intraventricular administration of *Tenebrio molitor* larvae extract regulates food intake and body weight in mice with high-fat diet–induced obesity. Nutr Res. (2017) 44:18–26. 10.1016/j.nutres.2017.05.011 28821314

[50] Hall F Johnson PE Liceaga A. Effect of enzymatic hydrolysis on bioactive properties and allergenicity of cricket (*Gryllodes sigillatus*) protein. Food Chem. (2018) 262:39–47. 10.1016/j.foodchem.2018.04.058 29751919

[51] Nongonierma AB Lamoureux C FitzGerald RJ. Generation of dipeptidyl peptidase iV (DPP-IV) inhibitory peptides during the enzymatic hydrolysis of tropical banded cricket (*Gryllodes sigillatus*) proteins. Food Funct. (2018) 9:407–16. 10.1039/C7FO01568B 29218344

[52] Zielińska E Karaś M Jakubczyk A. Antioxidant activity of predigested protein obtained from a range of farmed edible insects. Int J Food Sci Techn. (2017) 52:306–12. 10.1111/ijfs.13282

[53] Li X Xie H Chen Y Lang M Chen Y Shi L. Silkworm pupa protein hydrolysate induces mitochondria-dependent apoptosis and s phase cell cycle arrest in human gastric cancer sGC-7901 cells. Int J Mol Sci. (2018) 19:1013. 10.3390/ijms19041013 29597296 PMC5979490

[54] Gołebiowski M Urbanek A Oleszczak A Dawgul M Kamysz W Boguś MI. The antifungal activity of fatty acids of all stages of sarcophaga carnaria l. (*Diptera: sarcophagidae*). Microbiol Res. (2014) 169:279–86. 10.1016/j.micres.2013.07.011 23969191

[55] Cohn J Wat E Kamili A Tandy S. Dietary phospholipids, hepatic lipid metabolism and cardiovascular disease. Curr Opin Lipid. (2008) 19:257–62. 10.1097/MOL.0b013e3282ffaf96 18460916

[56] Zahradníčková H Tomčala A Berková P Schneedorferová I Okrouhlík J Šimek P. Cost effective, robust, and reliable coupled separation techniques for the identification and quantification of phospholipids in complex biological matrices: application to insects. J Separ Sci. (2014) 37:2062–8. 10.1002/jssc.201400113 24799084

[57] Mannarino MR Ministrini S Pirro M. Nutraceuticals for the treatment of hypercholesterolemia. Europ J Internal Med. (2014) 25:592–9. 10.1016/j.ejim.2014.06.008 24997485

[58] Pasha I Saeed F Waqas K Anjum FM Arshad MU. Nutraceutical and functional scenario of wheat straw. Crit Rev Food Sci Nutr. (2013) 53:287–95. 10.1080/10408398.2010.528080 23216000

[59] Zhang H Zheng H Chen J Chen XM Zhao H Zhang WW editors. Refined technique and characterization of Chinese insect wax. Appl Mech Mater. (2012) 161:94–9. 10.4028/www.scientific.net/AMM.161.94

[60] Ma J Ma L Zhang H Zhang Z Wang Y Li K. Policosanol fabrication from insect wax and optimization by response surface methodology. PLoS ONE. (2018) 13:e0197343. 10.1371/journal.pone.0197343 29763430 PMC5953464

[61] Brufau G Canela MA Rafecas M. Phytosterols: physiologic and metabolic aspects related to cholesterol-lowering properties. Nutr Res. (2008) 28:217–25. 10.1016/j.nutres.2008.02.003 19083411

[62] Ferretti G Bacchetti T Masciangelo S Bicchiega V. Effect of phytosterols on copper lipid peroxidation of human low-density lipoproteins. Nutrition. (2010) 26:296–304. 10.1016/j.nut.2009.04.015 19815390

[63] Koštál V Urban T Rimnáčová L Berková P Šimek P. Seasonal changes in minor membrane phospholipid classes, sterols and tocopherols in overwintering insect, pyrrhocoris apterus. J Insect Physiol. (2013) 59:934–41. 10.1016/j.jinsphys.2013.06.008 23845405

[64] Hahn T Roth A Febel E Fijalkowska M Schmitt E Arsiwalla T. New methods for high-accuracy insect chitin measurement. J Sci Food Agricult. (2018) 98:5069–73. 10.1002/jsfa.9044 29604075

[65] Kaya M Baran T Asan-Ozusaglam M Cakmak YS Tozak KO Mol A. Extraction and characterization of chitin and chitosan with antimicrobial and antioxidant activities from cosmopolitan orthoptera species (Insecta). Biotech Bioproc Eng. (2015) 20:168–79. 10.1007/s12257-014-0391-z

[66] Anderson JW Baird P Davis RH Ferreri S Knudtson M Koraym A. Health benefits of dietary fiber. Nutr Rev. (2009) 67:188–205. 10.1111/j.1753-4887.2009.00189.x 19335713

[67] Serna-Saldívar SO Gutiérrez-Uribe JA Mora-Rochin S García-Lara S. Potencial nutracéutico de los maíces criollos y cambios durante el procesamiento tradicional y con extrusión. Rev Fitotecnia Mexicana. (2013) 36:295–304. 10.35196/rfm.2013.3-S3-A.295

[68] Chen G Liu Y Zeng J Tian X Bei Q Wu Z. Enhancing three phenolic fractions of oats (Avena sativa l.) and their antioxidant activities by solid-state fermentation with *Monascus anka* and bacillus subtilis. J Cereal Sci. (2020) 93:102940. 10.1016/j.jcs.2020.102940

[69] Bunea A Rugina OD Pintea AM SconTA Z Bunea CI Socaciu C. Comparative polyphenolic content and antioxidant activities of some wild and cultivated blueberries from romania. Notulae Botanicae Horti Agrobot Cluj-Napoca. (2011) 39:70–6. 10.15835/nbha3926265

[70] Yoon Y-I Chung MY Hwang J-S Han MS Goo T-W Yun E-Y. *Allomyrina dichotoma* (Arthropoda: insecta) larvae confer resistance to obesity in mice fed a high-fat diet. Nutrients. (2015) 7:1978–91. 10.3390/nu7031978 25790040 PMC4377894

[71] Zhao X Song J-L Yi R Li G Sun P Park K-Y. Comparison of antioxidative effects of insect tea and its raw tea (Kuding tea) polyphenols in kunming mice. Molecules. (2018) 23:204. 10.3390/molecules23010204 29351230 PMC6017035

[72] Nongonierma AB FitzGerald RJ. Unlocking the biological potential of proteins from edible insects through enzymatic hydrolysis: a review. Innovat Food Sci Emerg Technol. (2017) 43:239–52. 10.1016/j.ifset.2017.08.014

[73] Han R Shin JT Kim J Choi YS Kim YW. An overview of the south korean edible insect food industry: challenges and future pricing/promotion strategies. Entomol Res. (2017) 47:141–51. 10.1111/1748-5967.12230

[74] Francardi V Frosinini R Pichini C Botta M Cito A Dreassi E. Galleria mellonella (*Lepidoptera pyralidae*): an edible insect of nutraceutical interest. REDIA. (2017) 3:285–294. 10.19263/REDIA-100.17.24

[75] Tzompa-Sosa DA Yi L van Valenberg HJF van Boekel MAJS Lakemond CMM. Insect lipid profile: aqueous vs. organic solvent-based extraction methods. Food Res Int. (2014) 62:1087–94. 10.1016/j.foodres.2014.05.052

[76] Haritos VS Horne I Damcevski K Glover K Gibb N. Unexpected functional diversity in the fatty acid desaturases of the flour beetle *tribolium castaneum* and identification of key residues determining activity. Insect Biochem Mol Biol. (2014) 51:62–70. 10.1016/j.ibmb.2014.05.006 24880119

[77] Harrabi S Boukhchina S Kallel H Mayer PM. Glycerophospholipid and triacylglycerol distribution in corn kernels (*Zea mays* l.). J Cereal Sci. (2010) 51:1–6. 10.1016/j.jcs.2009.04.013

[78] Küllenberg D Taylor LA Schneider M Massing U. Health effects of dietary phospholipids. Lipids Health Dis. (2012) 11:3. 10.1186/1476-511X-11-3 22221489 PMC3316137

[79] Leguizamón C Weller C Schlegel V Carr T. Plant sterol and policosanol characterization of hexane extracts from grain sorghum, corn and their dDGS. J Oil Fat Industries. (2009) 86:707–16. 10.1007/s11746-009-1398-z

[80] Marazzi G Cacciotti L Pelliccia F Iaia L Volterrani M Caminiti G. Long-term effects of nutraceuticals (berberine, red yeast rice, policosanol) in elderly hypercholesterolemic patients. Adv Ther. (2011) 28:1105–3. 10.1007/s12325-011-0082-5 22113535

[81] Fontani G Lodi L Migliorini S Corradeschi F. Effect of omega-3 and policosanol supplementation on attention and reactivity in athletes. J Am Coll Nutr. (2009) 28:473S–81S. 10.1080/07315724.2009.10718114 20234035

[82] Gabay O Sanchez C Salvat C Chevy F Breton M Nourissat G. Stigmasterol: a phytosterol with potential anti-osteoarthritic properties. Osteoarthr cartilage. (2010) 18:106–16. 10.1016/j.joca.2009.08.019 19786147

[83] Xiaoming C Ying F Hong Z Zhiyong C. Review of the nutritive value of edible insects. In: Durst PB Johnson DV Leslie RN Shono K, editors. Forest Insects as Food: Humans Bite Back. Proceedings of a Workshop on Asia-Pacific Resources and Their Potential for Development. Chiang Mai, Thailand. Food and Agriculture Organization of the United Nations (FAO). (2008) February 2008 pp.85–92 ref.28. Available online at: https://guides.lib.monash.edu/ld.php?content_id=48260115

[84] Leverstein-van Hall MA Dierikx CM Cohen Stuart J Voets GM Van Den Munckhof MP van Essen-Zandbergen A. Dutch patients, retail chicken meat and poultry share the same eSBL genes, plasmids and strains. Clin Microb Infect. (2011) 17:873–80. 10.1111/j.1469-0691.2011.03497.x 21463397

[85] Tiencheu B Womeni HM Linder M Mbiapo FT Villeneuve P Fanni J. Changes of lipids in insect (*Rhynchophorus phoenicis*) during cooking and storage. Europ J Lipid Sci Techn. (2013) 115:186–95. 10.1002/ejlt.201200284

[86] Bußler S Rumpold BA Jander E Rawel HM Schlüter OK. Recovery and techno-functionality of flours and proteins from two edible insect species: meal worm (*Tenebrio molitor*) and black soldier fly (*Hermetia illucens*) larvae. Heliyon. (2016) 2:e00218. 10.1016/j.heliyon.2016.e00218 28054035 PMC5198854

[87] Purschke B Tanzmeister H Meinlschmidt P Baumgartner S Lauter K Jäger H. Recovery of soluble proteins from migratory locust (*Locusta migratoria*) and characterisation of their compositional and techno-functional properties. Food Res Int. (2018) 106:271–9. 10.1016/j.foodres.2017.12.067 29579927

[88] Purschke B Meinlschmidt P Horn C Rieder O Jäger H. Improvement of techno-functional properties of edible insect protein from migratory locust by enzymatic hydrolysis. Europ Food Res Techn. (2018) 244:999–1013. 10.1007/s00217-017-3017-9

[89] Purschke B Sanchez YDM Jäger H. Centrifugal fractionation of mealworm larvae (*Tenebrio molitor*, l.) for protein recovery and concentration. LWT. (2018) 89:224. 10.1016/j.lwt.2017.10.057

[90] Zielińska E Karaś M Baraniak B. Comparison of functional properties of edible insects and protein preparations thereof. LWT. (2018) 91:168–74. 10.1016/j.lwt.2018.01.058

[91] van Huis A. Edible insects are the future? Proc Nutrit Soc. (2016) 75:294–305. 10.1017/S0029665116000069 26908196

[92] Ramos-Elorduy J. Threatened edible insects in hidalgo, mexico and some measures to preserve them. J Ethnobiol Ethnomed. (2006) 2:1–10. 10.1186/1746-4269-2-51 17144918 PMC1716161

[93] Sidali KL Pizzo S Garrido-Pérez EI Schamel G. Between food delicacies and food taboos: a structural equation model to assess western students' acceptance of amazonian insect food. Food Res Int. (2019) 115:83–9. 10.1016/j.foodres.2018.07.027 30599985

[94] Schlup Y Brunner T. Prospects for insects as food in switzerland: a tobit regression. Food Q Prefer. (2018) 64:37–46. 10.1016/j.foodqual.2017.10.010

[95] La Barbera F Verneau F Amato M Grunert K. Understanding westerners' disgust for the eating of insects: the role of food neophobia and implicit associations. Food Qual Prefer. (2018) 64:120–5. 10.1016/j.foodqual.2017.10.002

[96] Woolf E Zhu Y Emory K Zhao J Liu C. Willingness to consume insect-containing foods: a survey in the united states. LWT. (2019) 102:100–5. 10.1016/j.lwt.2018.12.010

[97] Lombardi A Vecchio R Borrello M Caracciolo F Cembalo L. Willingness to pay for insect-based food: the role of information and carrier. Food Qual Prefer. (2019) 72:177–87. 10.1016/j.foodqual.2018.10.001

[98] Jensen LD Miklos R Dalsgaard T Heckmann L-H Nørgaard J. Nutritional evaluation of common (*Tenebrio molitor*) and lesser (*Alphitobius diaperinus*) mealworms in rats and processing effect on the lesser mealworm. J Insects Food Feed. (2019) 5:1–10. 10.3920/JIFF2018.0048

[99] Hartmann C Siegrist M. Development and validation of the food disgust scale. Food Qual Prefer. (2018) 63:38–50. 10.1016/j.foodqual.2017.07.013 29496601

[100] Deroy O. Eat insects for fun, not to help the environment. Nat News. (2015) 521:395. 10.1038/521395 26017408

[101] Myers G Pettigrew S. A qualitative exploration of the factors underlying seniors' receptiveness to entomophagy. Food Res Int. (2018) 103:163–9. 10.1016/j.foodres.2017.10.032 29389602

[102] Farina MF. How method of killing crickets impact the sensory qualities and physiochemical properties when prepared in a broth. Int J Gastron Food Sci. (2017) 8:19–23. 10.1016/j.ijgfs.2017.02.002

[103] Chan EY. Mindfulness and willingness to try insects as food: the role of disgust. Food Qual Prefer. (2019) 71:375–83. 10.1016/j.foodqual.2018.08.014

[104] Magara HJO Niassy S Ayieko MA Mukundamago M Egonyu JP Tanga CM. Edible crickets (Orthoptera) around the world: distribution, nutritional value, and other benefits—a review. Front Nutr. (2021) 7:257. 10.3389/fnut.2020.537915 33511150 PMC7835793

[105] Shelomi M. Why we still don't eat insects: assessing entomophagy promotion through a diffusion of innovations framework. Trends Food Sci Techn. (2015) 45:311–8. 10.1016/j.tifs.2015.06.008

[106] Menozzi D Sogari G Veneziani M Simoni E Mora C. Eating novel foods: an application of the theory of planned behaviour to predict the consumption of an insect-based product. Food Qual Prefer. (2017) 59:27–34. 10.1016/j.foodqual.2017.02.001

[107] Fernandez-Cassi X Supenu A Jansson A Boqvist S Vagsholm I. Novel foods: a risk profile for the house cricket (*Acheta domesticus*). EFSA J. (2018) 5:137–57. 10.2903/j.efsa.2018.e16082 32626053 PMC7015497

[108] Kashiri M Marin C Garzon R Rosell C Rodrigo D Martínez A. Use of high hydrostatic pressure to inactivate natural contaminating microorganisms and inoculated *E. coli* O157:H7 on *Hermetia illucens* larvae. PLoS ONE. (2018) 13:e0194477. 10.1371/journal.pone.0194477 29566029 PMC5864016

[109] Marín C Urbina P Salvatierra P Rodrigo D Fernández P Martínez A. Effect of High Hydrostatic Pressure on natural contaminating microorganisms and inoculated Salmonella in *Hermetia illucens* larvae. Berl Münch Tierärztliche Wochenschrift. (2019) 131:257–63. 10.2376/0005-9366-18058

[110] Fasolato L Cardazzo B Carraro L Fontana F Novelli E Balzan S. Edible processed insects from e-commerce: food safety with a focus on the *Bacillus cereus* group. Food Microb. (2018) 76:296–303. 10.1016/j.fm.2018.06.008 30166154

[111] Osimani A Milanovic V Cardinali F Garofalo C Clementi F Pasquini M. The bacterial biota of laboratory-reared edible mealworms (*Tenebrio molito*r l.): from feed to frass. Int J Food Microb. (2018) 272:49–60. 10.1016/j.ijfoodmicro.2018.03.001 29525619

[112] Stoops J Crauwels S Waud M Claes J Lievens B Van Campenhout L. Microbial community assessment of mealworm larvae (*Tenebrio molitor*) and grasshoppers (*Locusta migratoria migratorioides*) sold for human consumption. Food Microb. (2016) 53:122–7. 10.1016/j.fm.2015.09.010 26678139

[113] Vandeweyer D Crauwels S Lievens B Van Campenhout L. Metagenetic analysis of the bacterial communities of edible insects from diverse production cycles at industrial rearing companies. Int J Food Microb. (2017) 261:11–8. 10.1016/j.ijfoodmicro.2017.08.018 28881263

[114] Klunder H Wolkers-Rooijackers JCM Korpela J Nout MJ. Microbiological aspects of processing and storage of edible insects. Food Control. (2012) 26:628–31. 10.1016/j.foodcont.2012.02.013

[115] Garofalo C Osimani A Milanovic V Taccari M Cardinali F Aquilanti L. The microbiota of marketed processed edible insects as revealed by high-throughput sequencing. Food Microbiol. (2017) 62:15–22. 10.1016/j.fm.2016.09.012 27889142

[116] Grabowski N Klein G. Microbiology of processed edible insect products – results of a preliminary survey. Int J Food Microb. (2016) 243:103–7. 10.1016/j.ijfoodmicro.2016.11.005 27903420

[117] Caparros Megido R Desmedt S Blecker C Béra F Haubruge E Alabi T. Microbiological load of edible insects found in belgium. Insects. (2017) 8:12. 10.3390/insects8010012 28098752 PMC5371940

[118] Ssepuuya G Wynants E Verreth C Crauwels S Lievens B Claes J. Microbial characterisation of the edible grasshopper *Ruspolia differens* in raw condition after wild-harvesting in uganda. Food Microbiol. (2018) 77:106–17. 10.1016/j.fm.2018.09.005 30297041

[119] Food Standards. Good Agricultural Practices for Cricket Farm. Acfs.go.th. (2018). Available online at: https://www.acfs.go.th/standard/download/eng/GAP_CRICKET_FARM-ENG.pdf (accessed May 23, 2020).

[120] Osimani A Garofalo C Milanovic V Taccari M Cardinali F Aquilanti L. Insight into the proximate composition and microbial diversity of edible insects marketed in the european union. Europ Food Res Techn. (2017) 243:1157–71. 10.1007/s00217-016-2828-4

[121] Grabowski N Klein G. Microbiology of cooked and dried edible mediterranean field crickets (*Gryllus bimaculatus*) and superworms (*Zophobas atratus*) submitted to four different heating treatments. Food Sci Techn Int. (2016) 23:17–23. 10.1177/1082013216652994 27235993

[122] Murefu TR Macheka L Musundire R Manditsera F. Safety of wild harvested and reared edible insects: a review. Food Control. (2019) 101:209–24. 10.1016/j.foodcont.2019.03.003

[123] Barre A Velazquez E Delplanque A Caze-Subra S Bienvenu F Bienvenu J. Les allergènes croisants des insectes comestibles. Rev Franç d'Allergologie et d'Immunologie Clinique. (2016) 56:522–32. 10.1016/j.reval.2016.02.027

[124] Sabolová M Kulma M Kourimská L. Sex-dependent differences in purine and uric acid contents of selected edible insects. J Food Compos Analy. (2021) 96:103746. 10.1016/j.jfca.2020.103746

[125] FAO. Looking at Edible Insects From a Food Safety Perspective. Challenges and Opportunities for the Sector. Rome. (2021). 10.4060/cb4094en

[126] EFSA Scientific Committee. Risk Profile Related To Production and Consumption of Insects as Food and Feed: Risk Profile of Insects as Food and Feed. Parma (2015). p. 13. 10.2903/j.efsa.2015.4257

[127] EFSA Panel on Nutrition NFaFAN. Scientific Opinion on the Safety of Yellow Mealworm (Tenebrio molitor larva) as a Novel Food Pursuant to Regulation (EU) 2015/2283. Parma (2021). p. 19. 10.2903/j.efsa.2021.6343

[128] Scientific Committee of the Federal Agency for the Safety of the Food Chain. (2014). Food Safety Aspects of Insects Intended for Human Consumption. SciCom dossier; 2014/04 no.9160 Avaliable online at: https://www.favv-afsca.be/scientificcommittee/opinions/2014/_documents/Advice14-2014_ENG_DOSSIER2014-04.pdf (accesaed Febuary 18, 2020).

[129] FAO. Regulation (EC) No. 999/2001 Laying Down Rules for the Prevention, Control and Eradication of Certain Transmissible Spongiform Encephalopathies. Rome: European Union (2001).

[130] Grabowski NT Tchibozo S Abdulmawjood A Acheuk F M'Saad Guerfali M Sayed WAA. Edible insects in africa in terms of food, wildlife resource, and pest management legislation. Foods. (2020) 9:502. 10.3390/foods9040502 32316132 PMC7230556

[131] KEBS. Production and Handling of Insects for Food and Feed— Code of Practice. Nairobi, Kenya (2020). Available online at: https://www.kebs.org/images/standards/public_review_standards/2020/June/DKS_2922_2_Products_containing_edible_insects_PR.pdf (accessed May 20, 2021).

[132] KEBS. Edible insects. Part *2. Products Containing Edible Insects— Specification*. (2020). Available online at: https://www.kebs.org/images/standards/public_review_standards/2020/June/DKS_2922_2_Products_containing_edible_insects_PR.pdf (accessed May 20, 2021).

[133] Lähteenmäki-Uutela A Grmelová N Hénault-Ethier L Deschamps MH Vandenberg G Zhao A. Insects as Food and Feed: Laws of the European Union, United States, Canada, Mexico, Australia, and China. JSTOR. (2017) 12:22–36. Available online at: http://www.jstor.org/stable/26451416

